# Construction of a novel model based on cell-in-cell-related genes and validation of KRT7 as a biomarker for predicting survival and immune microenvironment in pancreatic cancer

**DOI:** 10.1186/s12885-022-09983-6

**Published:** 2022-08-16

**Authors:** Jianlu Song, Rexiati Ruze, Yuan Chen, Ruiyuan Xu, Xinpeng Yin, Chengcheng Wang, Qiang Xu, Yupei Zhao

**Affiliations:** Department of General Surgery, State Key Laboratory of Complex Severe and Rare Diseases, Peking Union Medical College Hospital, Chinese Academy of Medical Sciences and Peking Union Medical College, Beijing, 100730 China

**Keywords:** Pancreatic cancer, Cell-in-cell, Prognostic model, Immune microenvironment, KRT7

## Abstract

**Background:**

Pancreatic cancer (PC) is a highly malignant tumor featured with high intra-tumoral heterogeneity and poor prognosis. Cell-in-cell (CIC) structures have been reported in multiple cancers, and their presence is associated with disease progression. Nonetheless, the prognostic values and biological functions of CIC-related genes in PC remain poorly understood.

**Methods:**

The sequencing data, as well as corresponding clinicopathological information of PC were collected from public databases. Random forest screening, least absolute shrinkage, and selection operator (LASSO) regression and multivariate Cox regression analysis were performed to construct a prognostic model. The effectiveness and robustness of the model were evaluated using receiver operating characteristic (ROC) curves, survival analysis and establishing the nomogram model. Functional enrichment analyses were conducted to annotate the biological functions. The immune infiltration levels were evaluated by ESTIMATE and CIBERSORT algorithms. The expression of KRT7 (Keratin 7) was validated by quantitative real-time PCR (qRT-PCR), western blotting and immunohistochemistry (IHC) staining. The CIC formation, cell clusters, cell proliferation, migration and invasion assays were applied to investigate the effects of silencing the expression of *KRT7*.

**Results:**

A prognostic model based on four CIC-related genes was constructed to stratify the patients into the low- and high-risk subgroups. The high-risk group had a poorer prognosis, higher tumor mutation burden and lower immune cell infiltration than the low-risk group. Functional enrichment analyses showed that numerous terms and pathways associated with invasion and metastasis were enriched in the high-risk group. *KRT7*, as the most paramount risk gene in the prognostic model, was significantly associated with a worse prognosis of PC in TCGA dataset and our own cohort. High expression of *KRT7* might be responsible for the immunosuppression in the PC microenvironment. *KRT7* knockdown was significantly suppressed the abilities of CIC formation, cell cluster, cell proliferation, migration, and invasion in PC cell lines.

**Conclusions:**

Our prognostic model based on four CIC-related genes has a significant potential in predicting the prognosis and immune microenvironment of PC, which indicates that targeting CIC processes could be a therapeutic option with great interests. Further studies are needed to reveal the underlying molecular mechanisms and biological implications of CIC phenomenon and related genes in PC progression.

**Supplementary Information:**

The online version contains supplementary material available at 10.1186/s12885-022-09983-6.

## Background

Pancreatic cancer (PC) is a highly lethal malignancy with rising an incidence and mortality worldwide, and it was estimated that PC will be the second leading cause of cancer-related death by 2030 [1, 2]. Currently, curative surgery followed by adjuvant chemotherapy remains a standard therapeutic approach, however, because of the concealed anatomical location of the pancreas, dormant symptoms, and deficiency of reliable biomarkers, effective screening is not available for PC, and most patients present with locally advanced or metastatic disease at the time of initial diagnosis, leading to missed opportunities for surgical intervention [3]. Therefore, clarifying the mechanisms of PC progression and developing more effective therapeutic strategies is essential.

Cell-in-cell (CIC) structures refer to the presence of one or more living cells internalized into another living one with the formation of “bird-eye cells”, which was first reported approximately 120 years ago in tumor tissues [4]. CIC is a long-standing phenomenon virtually neglected for decades but has attracted great interest in recent years. CIC structures have been observed in various types of tumors and are associated with a worse prognosis, such as breast cancer, lung cancer, and PC [5–7]. From cellular mechanisms, cell cannibalism and entosis are two of the best-characterized CIC processes in cancers [8]. Cell cannibalism generally refers to the engulfment of live or dead cells within cancer cells through a mechanism involving actin, ezrin, and caveolin. Previous studies have reported that cancer cells exert potent engulfment activity directed toward homotypic cancer cells, lymphocytes, neutrophils, natural killer cells, and mesenchymal stem cells [4, 9–11]. Entosis is a specific form of cell cannibalism mainly induced by extracellular matrix detachment, aberrant mitosis, and glucose starvation [12], which is thought to occur primarily between homotypic epithelial cells following the establishment of adherent junctions via E-cadherin or P-cadherin, formation of mechanical ring enriched in vinculin and actomyosin contraction mediated by the Rho − ROCK − DIAPH1 signaling pathway [13]. Unlike cell cannibalism engulfed by outside cells, the internalized cells actively penetrate into outside cells driving by contractile actomyosin at the opposite cell cortex and subsequently die by lysosome-dependent degradation in entosis, leading to a non-apoptotic cell death [14]. In the past decade, numerous studies have shown that cannibalistic behavior is a hallmark of cancer, conferring cancer cells metabolic advantages under starvation [8, 9, 15]. Moreover, researchers have shown that entosis can promote direct competition among cancer cells in mixed populations and ploidy changes of outside cells, affecting the clonal selection and evolution of cancer cells [16, 17]. A recent study reported that in pancreatic ductal adenocarcinoma (PDAC), the most common type of PC, CIC structures were more prevalent in liver metastasis than the primary tumor and poorly differentiated adenocarcinoma or adenosquamous carcinoma than well or moderately differentiated adenocarcinoma, suggesting that CIC phenomenon is associated with the aggressive biology of PC [7]. Therefore, evaluating the CIC status is a powerful method for prognosis estimation. However, only few studies focused on the prognostic value of CIC-related genes and their biological functions in PC.

In the present study, we systematically analyzed the expression profiles and prognostic values of CIC-related genes using public datasets. A corresponding CIC-related signature was identified and estimated, and the functional enrichment analyses were performed, and somatic mutation profiles and immune features were compared between the low- and high-risk subgroups to explore the potential mechanisms. Our results demonstrated that the high-risk group showed poorer prognosis, higher somatic mutation frequencies, and lower immune infiltration levels than the low-risk group. Furthermore, *KRT7* (Keratin 7) was identified as the most paramount risk gene with its high expression being significantly associated with a worse prognosis and immunosuppression in PC. As validated in our own independent cohort, KRT7 is an unfavorable marker of PC, and its high expression positively is correlated with CIC formation, cell cluster, cell proliferation, migration, and invasion in vitro. The results of this study may help to improve the current plight of PC treatment by designing combined therapeutic strategies targeting CIC-related processes.

## Methods

### Datasets and processing

The RNA sequencing (RNA-seq) data of the Genotype-Tissue Expression (GTEx) and The Cancer Genome Atlas (TCGA) datasets were downloaded from the University of California Santa Cruz (UCSC) Xena website (https://xenabrowser.net/datapages/), which included 167 normal samples and 179 tumor samples. The expression data were normalized to transcripts per million (TPM) values and transformed to log_2_(TPM + 1). The RNA-seq data of the International Cancer Genome Consortium (ICGC) dataset were also downloaded from UCSC Xena website, which included 96 tumor samples. Its expression data were normalized to counts per million (CPM) and transformed to log_2_(CPM). The normalized expression matrix from microarray datasets of GSE21501, GSE62452, and GSE71729 were downloaded from the Gene Expression Omnibus (GEO) database (http://www.ncbi.nlm.nih.gov/geo/), which included 132, 69, and 145 tumor samples, respectively. The corresponding clinicopathological information and somatic mutation data of TCGA and ICGC datasets were obtained from TCGA (http://portal.gdc.cancer.gov/) and ICGC (http://dcc.icgc.org/) official websites. By combining the expression data with corresponding clinicopathological information, samples with absent information of survival status, age, sex, grade, TNM stage, and patients lost to follow-ups or follow-up time less than 30 days were excluded. Meanwhile, two samples from GTEx dataset were eliminated due to the low expression levels (less 10,000 genes). Finally, GTEx (165 normal samples) and TCGA (167 tumor samples) datasets were chosen as the training cohort. ICGC (87 samples), GSE21501 (98 samples), GSE62452 (64 samples), and GSE71729 (123 samples) datasets were applied for external validation. The clinical characteristics of patients are shown in Table 1. The microarray and RNA-seq data of PC cell lines were obtained from GSE21654, GSE40098 and the Cancer Cell Line Encyclopedia (CCLE) database (https://sites.broadinstitute.org/ccle/). The processed single-cell RNA sequencing (scRNA-seq) data of the CRA001160 dataset (including over 57,000 cells from 24 primary PDAC samples and 11 normal samples) were downloaded from the National Genomics Data Center (NGDC) database (https://ngdc.cncb.ac.cn/).

**Table1:** Clinical characteristics of pancreatic cancer patients in the multiple datasets

Variables	TCGA	ICGC	GSE21501	GSE62452	GSE71729
	*n* = 167 (%)	*n* = 87 (%)	*n* = 98 (%)	*n* = 64 (%)	*n* = 123 (%)
**Age**					
< 65	77 (46.1)	35 (40.2)	NA	NA	NA
≥ 65	90 (53.9)	52 (59.8)	NA	NA	NA
**Gender**					
Female	76 (45.5)	41 (47.1)	NA	NA	NA
Male	91 (54.5)	46 (52.9)	NA	NA	NA
**Grade**					
G1 − G2	118 (70.7)	54 (62.1)	NA	33 (51.6)	NA
G3 − G4	49 (29.3)	33 (37.9)	NA	31 (48.4)	NA
**Stage**					
I − II	160 (95.8)	NA	NA	48 (75.0)	NA
III − IV	7 (4.2)	NA	NA	16 (25.0)	NA
**T**					
T1 − T2	27 (16.2)	13 (14.9)	18 (18.4)	NA	NA
T3 − T4	140 (83.8)	74 (85.1)	80 (81.6)	NA	NA
**N**					
N0/NX	49 (29.3)	28 (32.2)	12 (12.2)	NA	NA
N1	118 (70.7)	59 (67.8)	86 (87.8)	NA	NA
**Subtype**					
Immunogenic	NA	22 (25.3)	NA	NA	NA
ADEX	NA	14 (16.1)	NA	NA	NA
Progenitor	NA	29 (33.3)	NA	NA	NA
Squamous	NA	22 (25.3)	NA	NA	NA
Classical	NA	NA	NA	NA	88 (71.5)
Basal-like	NA	NA	NA	NA	35 (28.5)
**Status**					
Alive	76 (45.5)	32 (36.8)	35 (35.7)	15 (23.4)	40 (32.5)
Dead	91 (54.5)	55 (63.2)	63 (64.3)	49 (76.6)	83 (67.5)

*TCGA* The Cancer Genome Atlas, *ICGC* International Cancer Genome Consortium, *ADEX* Aberrantly Differentiated Endocrine Exocrine, *NA* not available

### Identification of differentially expressed genes (DEGs)

A total of 101 CIC-related genes were extracted from GeneCards website (http://www.genecards.org/) and previous literature [4, 8, 12, 14], searching by the keywords “cell-in-cell”, “cell cannibalism”, and “entosis” (detailed in Table S1**)**. The “limma” R package was used to identify the DEGs between normal and tumor samples. False discovery rate (FDR) < 0.05 and |log_2_(Fold Change)|≥ 1 were determined as the significance criteria for selecting DEGs, same for the identification of the CIC-related DEGs between two risk groups. The results of DEGs were visualized by volcano plots and heatmaps.

### Establishment and verification of the cic-related prognostic model

The random forest screening (using “randomForest” R package) and least absolute shrinkage and selection operator (LASSO) regression analysis (using “glmnet” R package) were applied to screen important DEGs in TCGA cohort. And then, the overlapping CIC-related DEGs between these two methods were further selected to construct the best regression model via a stepwise multivariate Cox regression analysis. Finally, the risk score of each patient was calculated by the regression coefficient of each gene in the prognostic model and corresponding expression level using the following formula: Risk score = (Expr_gene1_ × Coef_gene1_) + (Expr_gene2_ × Coef_gene2_) + … + (Expr_genen_ × Coef_genen_). For external validation cohorts, the risk score of each patient was calculated by the above formula. Patients were stratified into the low- and high-risk subgroups according to the median value of risk scores. Principal component analysis (PCA) was applied to visualize the clustering conditions of the prognostic signature. The Kaplan–Meier survival analysis was used to compare the differences in overall survival (OS) probability between two groups, and time-dependent receiver operating characteristic (ROC) curves were used to evaluate the predictive accuracy of the prognostic model. The univariate and multivariate Cox regression analyses were performed to determine the independent prognostic factors associated with OS. The nomogram was established to predict the 1-, 2-, 3-year survival probability based on the risk score and other clinicopathological characteristics. The corresponding C-index, time-dependent ROC curves, calibration curves, and decision curve analysis (DCA) were drawn to assess the efficiency of the nomogram. The Human Protein Atlas (HPA) database (http://www.proteinatlas.org) was used to investigate the expression of signature-related genes in normal pancreas and PC tissues from protein levels.

### Functional enrichment analysis for DEGs

Based on Gene Ontology (GO) and Kyoto Encyclopedia of Genes and Genomes (KEGG) databases [18], functional enrichment analyses of DEGs were conducted to clarify the biological functions of genes by applying the “clusterProfiler” R package [19]. GeneMANIA (http://genemania.org/), a platform for gene prioritization and predicting gene function, was used to predict the related networks and functions of CIC-related genes [20].

### Somatic mutation analysis and immune feature estimation

The landscape of somatic mutations was analyzed and visualized via the “maftools” R package. Frequencies of somatic mutations and tumor mutation burdens (TMB) were calculated and compared between the low- and high-risk groups. The estimate score, stromal score, immune score, and tumor purity were calculated by the ESTIMATE algorithm [21]. The CIBERSORT algorithm was performed to quantify the relative abundance of 22 immune cells infiltrated in tumor microenvironment (TME) [22]. The immune subtypes of individuals were classified by using the “ImmuneSubtypesClassifier” R package. Representative immune checkpoints were extracted from previous literatures and the expression levels of them were compared. The immunotherapy responses of individuals were analyzed by ImmuCellAI website (http://bioinfo.life.hust.edu.cn/ImmuCellAI) [23].

### Clinical specimens and immunohistochemical analysis

A total of 55 patients with primary PDAC who underwent surgical resection at the Peking Union Medical College Hospital (PUMCH) were recruited in this study strictly following the guidelines of the Ethics Committee of Peking Union Medical College Hospital, and written informed consent was obtained from all patients. The tumor and adjacent normal tissues were fixed by 10% formalin and embedded by paraffin. The sections of tissue specimens were used for immunohistochemistry (IHC) incubated with antibody against KRT7 (1:2000; Proteintech, #17,513–1-AP). Manual staining and the estimation of IHC score were performed as previously described [24, 25].

### scRNA-seq data quality control, dimension reduction, and cell clustering

The raw data of scRNA-seq were processed previously [26], and the merged dataset was imported into the Seurat (v4.0.6) R toolkit for quality control and further analysis [27]. Low quality cells (< 200 genes/cell, < 3 cells/gene and > 10% mitochondrial genes) were removed. The gene expression profiles were then normalized using the “NormalizeData” function, and the top 2000 highly variable genes were generated with the “FindVariableFeatures” function to perform PCA. Significantly principal components were determined using JackStraw analysis. The “FindNeighbors” and “FindClusters” functions were used to perform cell clustering. The single-cell clustering was visualized via the uniform manifold approximation and projection (UMAP) analysis using the “RunUMAP” function. We annotated the cell types of cell clusters using the cellular markers provided by the original authors. Cells identified with malignant fractions were subjected to re-cluster by the above functions, and the dot plot was used to display the relative expression of genes among diverse malignant cell clusters.

### Cell culture

Four PC cell lines BxPC-3, CFPAC-1, PANC-1, and MIA PaCa-2 were purchased from the American Type Culture Collection (ATCC, Manassas, VA, USA). All cell lines were regularly tested for Mycoplasma and identified by Short Tandem Repeat (STR) identification. BxPC-3 cell line was cultured in RPMI-1640 medium (Corning, #10–040-CV). CFPAC-1 cell line was cultured in Iscove’s Modified Dulbecco Medium (IMDM; Corning, #15–016-CV). PANC-1 and MIA PaCa-2 cell lines were cultured in high glucose Dulbecco’s Modified Eagle Medium (DMEM; Corning, #10–013-CMR). All medium was supplemented with 10% fetal bovine serum (HyClone, #SH30073.03) and 1% Penicillin–Streptomycin (Life Technologies, #15,140–122). Cells were routinely maintained at 37℃ with 5% CO_2_.

### RNA Extraction and quantitative real-time PCR analysis

Total RNA was extracted from cultured cells by the TRIzol reagent (Life Technologies, #15,596–026) and cDNA synthesis was performed using the RevertAid First Strand cDNA Synthesis Kit (Thermo Scientific™, #K1622) following the manufacturer’s instructions. Quantitative real-time PCR (qRT-PCR) was performed in triplicate using SYBR Green Master Mix (Applied Biosystems, #A25742) [24]. The expression levels of *GAPDH* were used as the endogenous control and the relative expression of *KRT7* was calculated using the 2^−ΔΔCt^ method. The sequences of the primers used for qRT-PCR are as follows:

*KRT7*: Forward 5’- CGAGGATATTGCCAACCGCAG-3’,

Reverse 5’-CCTCAATCTCAGCCTGGAGCC-3’;

*GAPDH*: Forward 5’-GTCTCCTCTGACTTCAACAGCG-3’,

Reverse 5’- ACCACCCTGTTGCTGTAGCCAA-3’.

### Western blotting

Protein extracts from cells were prepared using 2% SDS lysis buffer including protein phosphatase inhibitor (Thermo Scientific™, #78,440). Total protein (20 μg) was subjected to 10% (v/v) SDS-PAGE gels and transferred to nitrocellulose filter membrane (Pall, #P-N66485). After blocking with 5% bovine serum albumin (BSA) for 2 h at 37 ℃, the membranes of proteins with different molecular weight (KDa) were cut according to the location of the markers (GenStar, M222) with enough spaces left at the edges of the first and the last sample lanes, then the cropped membranes were incubated with primary antibodies at 4℃ overnight. The primary antibodies anti-KRT7 (1:1000; Proteintech, #17,513–1-AP), anti-E-cadherin (1:5000; Proteintech, #20,874–1-AP), anti-P-cadherin (1:1000; Proteintech, #13,773–1-AP), anti-RHOA (1:1000; Proteintech, #10,749–1-AP), anti-Phospho-MLC2 (1:1000; Cell Signaling Technology, #3671), anti-β-Actin (1:20,000; Proteintech, #66,009–1-lg) and anti-GAPDH (1:50,000; Proteintech, #60,004–1-lg) were used and then incubated with HRP-conjugated secondary antibodies (1:5000; Proteintech, #SA00001-1 and # SA00001-2) at room temperature for 1 h and imaged through ECL kit (Beyotime, #P0018AM).

### CIC Formation and cell cluster assays

CIC formation assay was performed as previously described [13]. Briefly, about 2.0 × 10^5^ cells were suspended in a six-well plate precoated with 1 mL solidified 0.5% soft agar for 8 h and then mounted onto glass slides by Cytospin preparation. Cells were fixed by 4% paraformaldehyde solution and immunostained with Phalloidin (1:1000; Abcam, #ab176753), anti-KRT7 (1:200; Proteintech, #17,513–1-AP), CoraLite594-conjugated Goat Anti-Rabbit secondary antibody (1:200; Proteintech, #SA00013-4), and DAPI (Abcam, #ab104139). Images were acquired by Nikon AX / AX R confocal microscope. Structures with more than half of cell internalized were counted as CIC structures. One cell cluster was defined as cell colony that contains six or more cells and cells in cluster rate (%) = (number of cells involved in all clusters / number of total cells) × 100% [28].

### siRNA Transient transfection

Short interference RNAs (siRNAs) for *KRT7* were designed and chemically synthesized by RiboBio (RiboBio, Guangzhou, China). Sequences of siRNAs used in this study were as follows: siKRT7 1#: GTGGGAGCCGTGAATATCT; siKRT7 2#: GCCTCCCAGACATCTTTGA. For cell transfection, 5.0 × 10^5^ cells were transfected with 50 nM (BxPC-3 cells) or 150 nM (CFPAC-1 cells) siRNA using Lipofectamine 3000 (Invitrogen, Carlsbad, CA, USA) according to the manufacturer’s instruction. At 24 h or 48 h post-transfection, PC cells were harvested for functional experiments and total protein was extracted at 72 h post-transfection.

### Proliferation assays

Cell Counting Kit-8 (CCK-8) reagent (Dojindo Laboratories, #CK04) was used to measure cell proliferation. Transfected cells were seeded into 96-well plates (3,000 cells/well), and the medium of each well was replaced with 100 μL serum-free medium (including 10 μL CCK-8 solution) at 0, 24, 48, 72, 96 h after adherence. The optical density (OD) values were measured at a wavelength of 450 nm using the microplate reader after incubating for 2 h.

### Cell migration and invasion assays

The migrative and invasive abilities of transfected cells were evaluated using Transwell chambers (24-well and 8.0 μm pore size, Corning, #3422). For invasion assays, the chambers were precoated with Matrigel (Corning, #354,234), and 1.0 × 10^5^ transfected cells resuspended in 150 μL serum-free medium were seeded into the upper chambers and 600 μL supplemented medium with 10% FBS was added to the lower chambers. After incubating for 24 h (BxPC-3 cell line) or 36 h (CFPAC-1 cell line), the migrated or invaded cells were fixed with methanol and then stained with 0.5% crystal violet for 20 min at room temperature. The cells in five random fields (at × 200 magnification) per chamber were counted.

### Statistical analysis

Statistical analyses and visualization were performed using R (version 4.1.0) software and GraphPad Prism 9 (version 9.3.0). Kaplan–Meier analysis and log-rank test were used to evaluate the associations with survival time. Student’s t test, Mann–Whitney test, and chi-square test or Fisher’s exact test were utilized for the comparisons between two groups. Pearson’s correlation was used to assess the linear relationships between two genes. Biological replicates are shown as means ± standard deviation (SD). All *P* values of statistical results were based on two-sided statistical tests, and a *P* value < 0.05 was considered statistically significant.

## Results

### Differential gene expression analysis and functional enrichment analysis of CIC-related genes

**Fig. 1:**
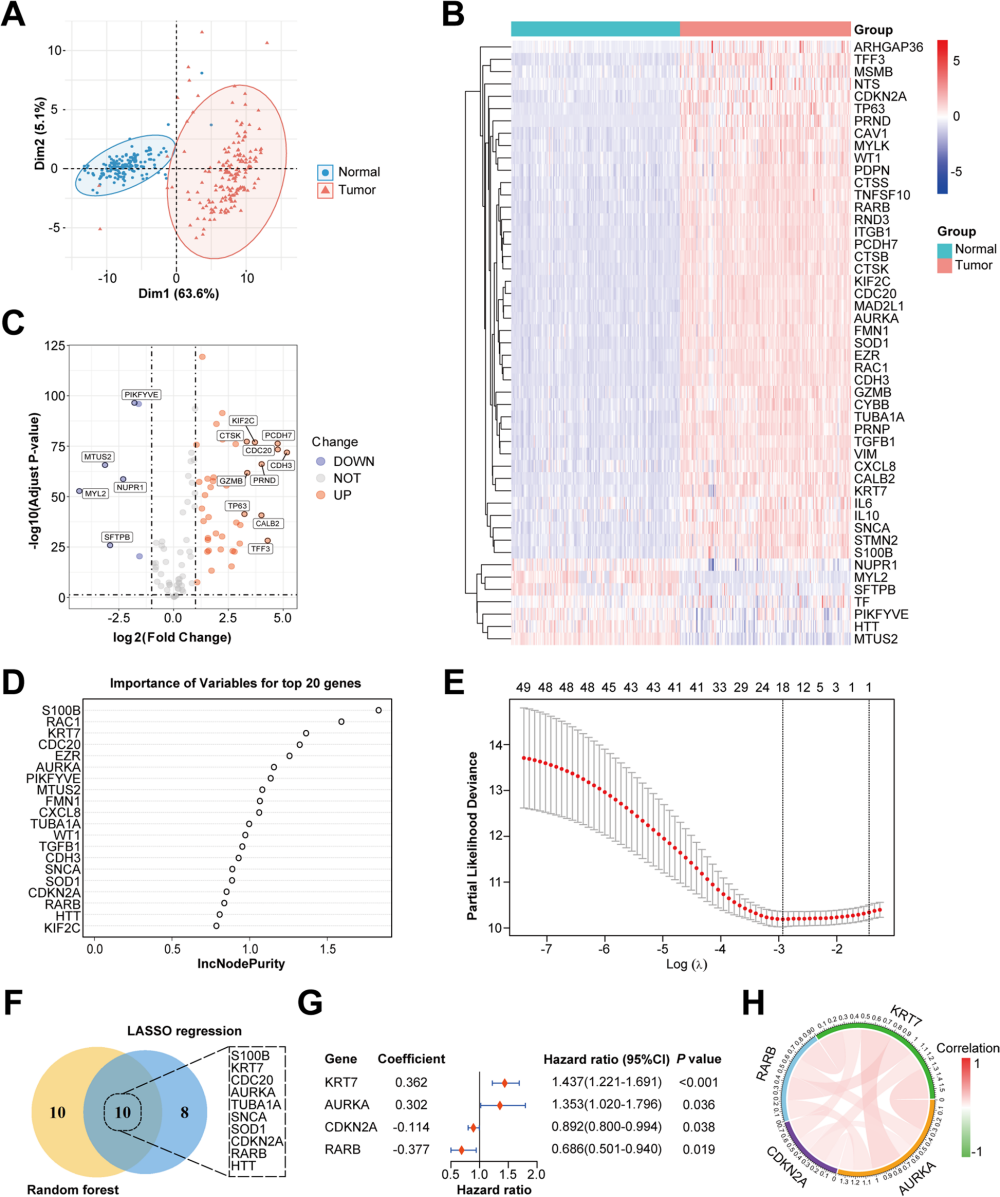
Identification of DEGs and construction of the CIC-related prognostic signature. **A** PCA based on CIC-related genes of tumor and normal samples from the TCGA and GTEx datasets. **B **and** C** Heatmap and volcano plot of CIC-related DEGs between normal and tumor samples. **D** Top 20 genes sorted by importance of variables using random forest screening. **E** The most proper log (λ) value in LASSO regression analysis. **F** Ten overlapping genes based on the results of random forest screening and LASSO regression analysis. **G** The results of multivariate Cox regression analysis for 4 significantly CIC-related genes contributing to OS in PC. **H** The correlation analysis of the 4 genes

The expression levels of 101 CIC-related genes were explored in normal and PC samples using GTEx and TCGA datasets. PCA showed that the distribution differs between normal and tumor samples (Fig. 1A). A total of 49 DEGs were identified, including 42 upregulated and 7 downregulated genes, and visualized by the heatmap (Fig. 1B) and volcano plot (Fig. 1C). GO analysis suggested that these DEGs were mainly involved in reactive oxygen species metabolic process, receptor-mediated endocytosis, cell leading edge, endocytic vesicle, tubulin binding and cytokine activity (Fig. S1A). Moreover, KEGG pathway analysis indicated that apoptosis, phagosome, regulation of actin cytoskeleton, ferroptosis, transcriptional dysregulation in cancer and focal adhesion were enriched (Fig. 1B).

### Construction of the CIC-related prognostic model in PC

To reduce the number of genes needed for constructing the prognostic model, we first utilized random forest screening to assign an importance factor to each CIC-related DEGs. And then, top 20 genes, ranked by importance, were selected for further analysis (Fig. 1D). Meanwhile, LASSO regression analysis was performed on 49 DEGs, and 18 candidate genes were retained by the most proper value of lambda (λ) (Fig. 1E). Subsequently, we combined 10 overlapping candidate genes between these two methods to establish the best regression model by a stepwise multivariate Cox regression analysis (Fig. 1F). Finally, four CIC-related genes significantly contributing to OS in PC patients were confirmed (Fig. 1G), and the risk score of each patient was calculated using the following formula: Risk score = (0.362 × expression level of *KRT7*) + [0.302 × expression level of *AURKA* (Aurora Kinase A)] + [− 0.114 × expression level of *CDKN2A* (Cyclin Dependent Kinase Inhibitor 2A)] + [− 0.377 × expression level of *RARB* (Retinoic Acid Receptor Beta)]. The correlation analysis among these four genes was shown in the circle plot (Fig. 1H). Moreover, the related networks and functions were predicted using GeneMANIA website (Fig. S1C). We found that related functions mainly involved in the regulation of cell cycle, mitosis-related process and kinase regulator activity.

### Evaluation and validation of the CIC-related prognostic model

**Fig. 2:**
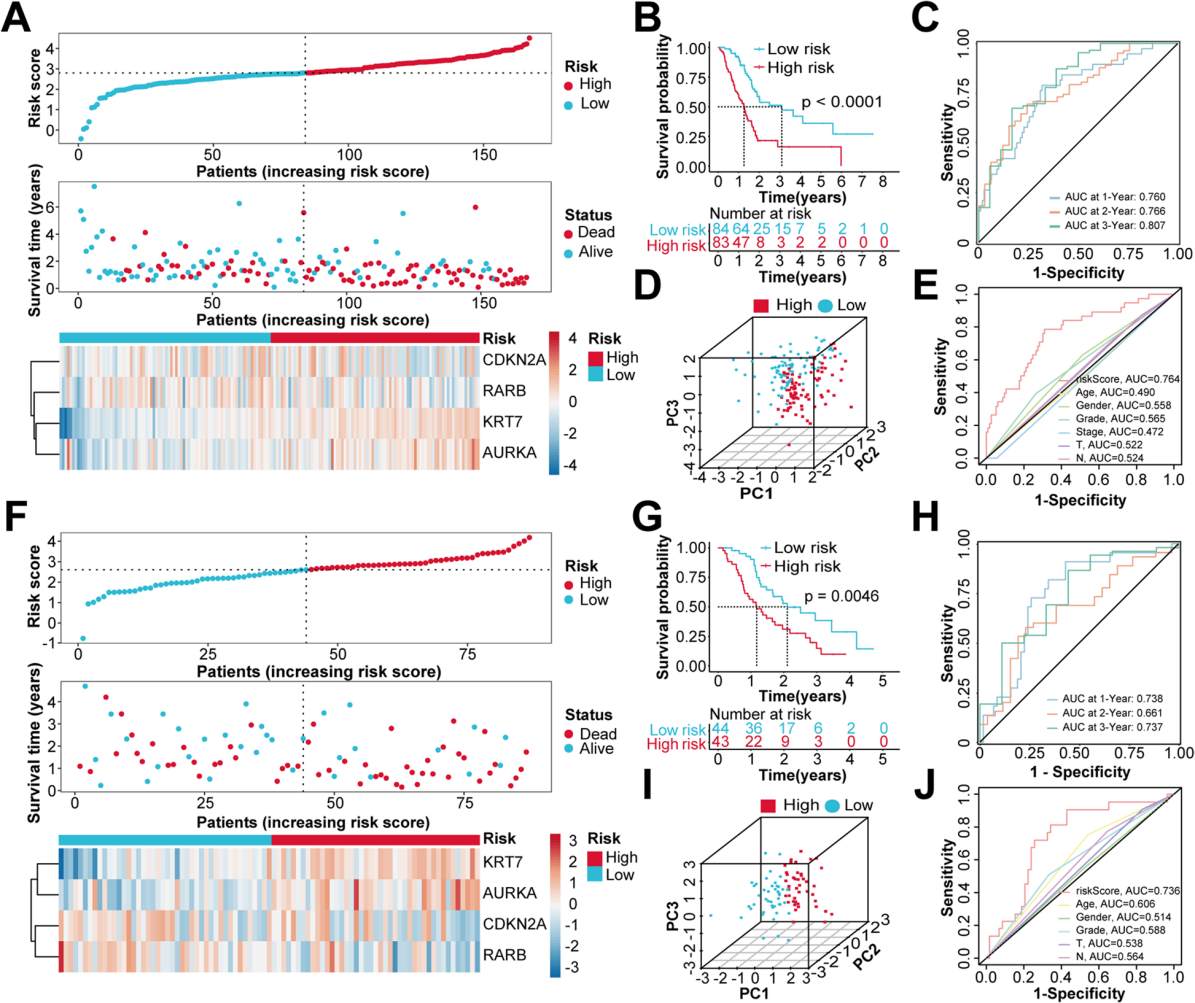
Evaluation and validation of CIC-related prognostic signature in TCGA and ICGC cohorts. **A **and **F** Distribution of risk scores, OS status overview, and heatmaps of four genes expression in TCGA (A) and ICGC (F) cohorts. **B **and **G** Kaplan–Meier curves for OS of patients between the low- and high-risk groups in TCGA (B) and ICGC (G) cohorts. **C **and** H** ROC curves for 1-, 2- and 3-year OS prediction of the prognostic signature in TCGA (C) and ICGC (H) cohorts. **D **and** I** PCA based on the prognostic signature in TCGA (D) and ICGC(I) cohorts. **E **and **J** ROC curves of the risk score and other clinicopathological characteristics in TCGA (E) and ICGC (J) cohorts

Based on the median of risk scores, patients in TCGA cohort were separated into the low- and high-risk groups. The scatterplots showed that, as the patient’s risk score increased, the number of deaths increased, and the survival time decreased. Compared to the low-risk group, the expression levels of *KRT7* and *AURKA* in the high-risk group were upregulated, while the expression levels of *CDKN2A* and *RARB* were downregulated (Fig. 2A). The Kaplan–Meier survival analysis indicated that patients in the high-risk group have a shorter OS than those in the low-risk group (Fig. 2B). The PCA revealed that patients with different risk levels were distributed into two clusters (Fig. 2D). The area under curves (AUC) were 0.760, 0.766 and 0.807 in the 1-year, 2-year, and 3-year ROC curves, respectively (Fig. 2C). We also found that, compared with other clinicopathological features, the AUC value of risk score was much higher, suggesting that it was a better prognostic indicator for PC patients (Fig. 2E). To demonstrate the robustness of the prognostic signature, the predictive efficiency was evaluated in three independent validation cohorts, including ICGC, GSE21501 and GSE62452. The patients from these three validation cohorts were stratified into the low- and high-risk groups based on the median of risk scores calculated by using the same formula as in TCGA modeling cohort. Consistently, patients in the high-risk group demonstrated a worse prognosis than the low-risk group. Similarly, the expression levels of *KRT7* and *AURKA* in the high-risk group were increased, while the expression levels of *CDKN2A* and *RARB* were decreased (Fig. 2F and 2G, Fig. S2A, B, F and G). The PCA confirmed that patients in different subgroups could be divided into two separate directions (Fig. 2I, Fig. S2D and I). Moreover, the AUC value of time-dependent ROC curves analysis reached around 0.700, indicating that the prognostic model performed well in three validation cohorts (Fig. 2H, Fig. S2C and H), and the risk score had better predictive accuracy compared to other clinicopathological characteristics (Fig. 2J, Fig. S2E and J). The univariate and multivariate Cox regression analyses were performed to evaluate the prognostic power of the risk signature. Based on the multivariate Cox regression analysis, the risk score was confirmed to be an independent prognostic factor for OS prediction in all four cohorts (Table S2).

### Establishment and validation of a predictive nomogram based on the risk signature

**Fig. 3:**
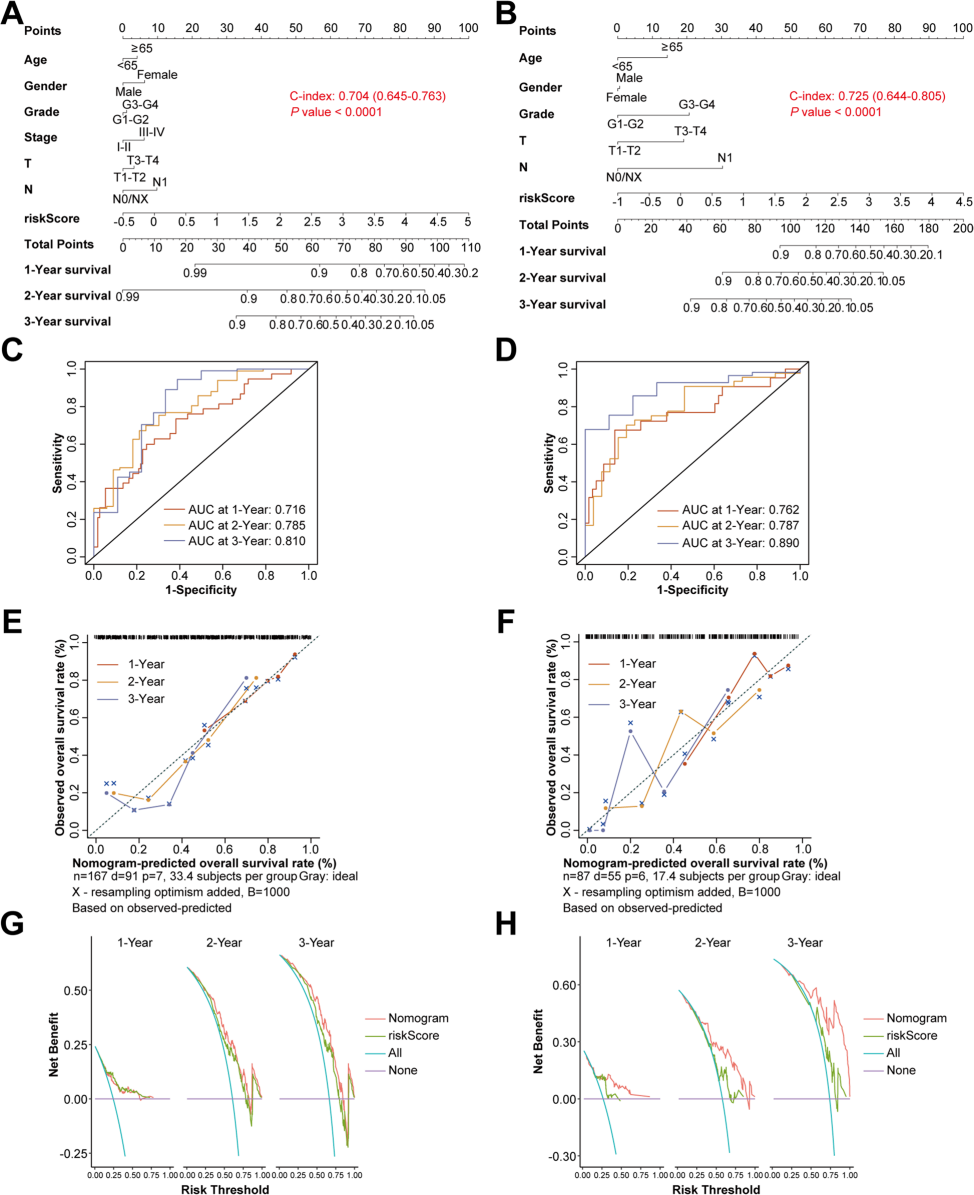
Establishment and evaluation of the predictive nomogram model. **A **and** B** Nomograms based on the risk score and clinicopathological characteristics for predicting the probability of 1-, 2-, 3-year OS in TCGA (A) and ICGC (B) cohorts. **C **and **D **Time-dependent ROC analysis of the nomogram in TCGA (C) and ICGC (D) cohorts. **E **and **F** Calibration curves of the nomogram in terms of agreement between observed and predicted 1-, 2- and 3-year survival probability in TCGA (E) and ICGC (F) cohorts. **G **and **H** The 1-, 2- and 3-year DCA curves of the nomogram in TCGA (E) and ICGC (F) cohorts

To further improve the predictive efficiency, the risk score and other clinicopathological characteristics such as age, sex, grade, and TNM stage were used to establish the predictive nomogram in TCGA and ICGC cohorts altogether. The C-index for the nomogram was 0.704 (95%CI 0.645 − 0.763) in TCGA cohort and 0.725 (95%CI 0.644 − 0.805) in ICGC cohort, indicating that the two nomograms both had well predictive performance (Fig. 3A and B). Subsequently, the time-dependent ROC curves, calibration curves and DCA were applied to further evaluate the effectiveness of established nomograms. The AUCs of ROC curves for predicting 1-, 2-, and 3-year survival were 0.716, 0.785 and 0.810 in TCGA cohort (Fig. 3C), 0.762, 0.787 and 0.890 in ICGC cohort (Fig. 3D). In addition, the calibration curves presented satisfied coherence between observed and predicted 1-year, 2-year and 3-year OS in both cohorts (Fig. 3E and F). The results of DCA demonstrated that the nomogram achieved the highest net benefits, suggesting that it was an efficient model to predict the prognosis of PC patients (Fig. 3G and H).

### Functional enrichment analyses of DEGs and somatic mutation profiles between two risk groups

**Fig. 4:**
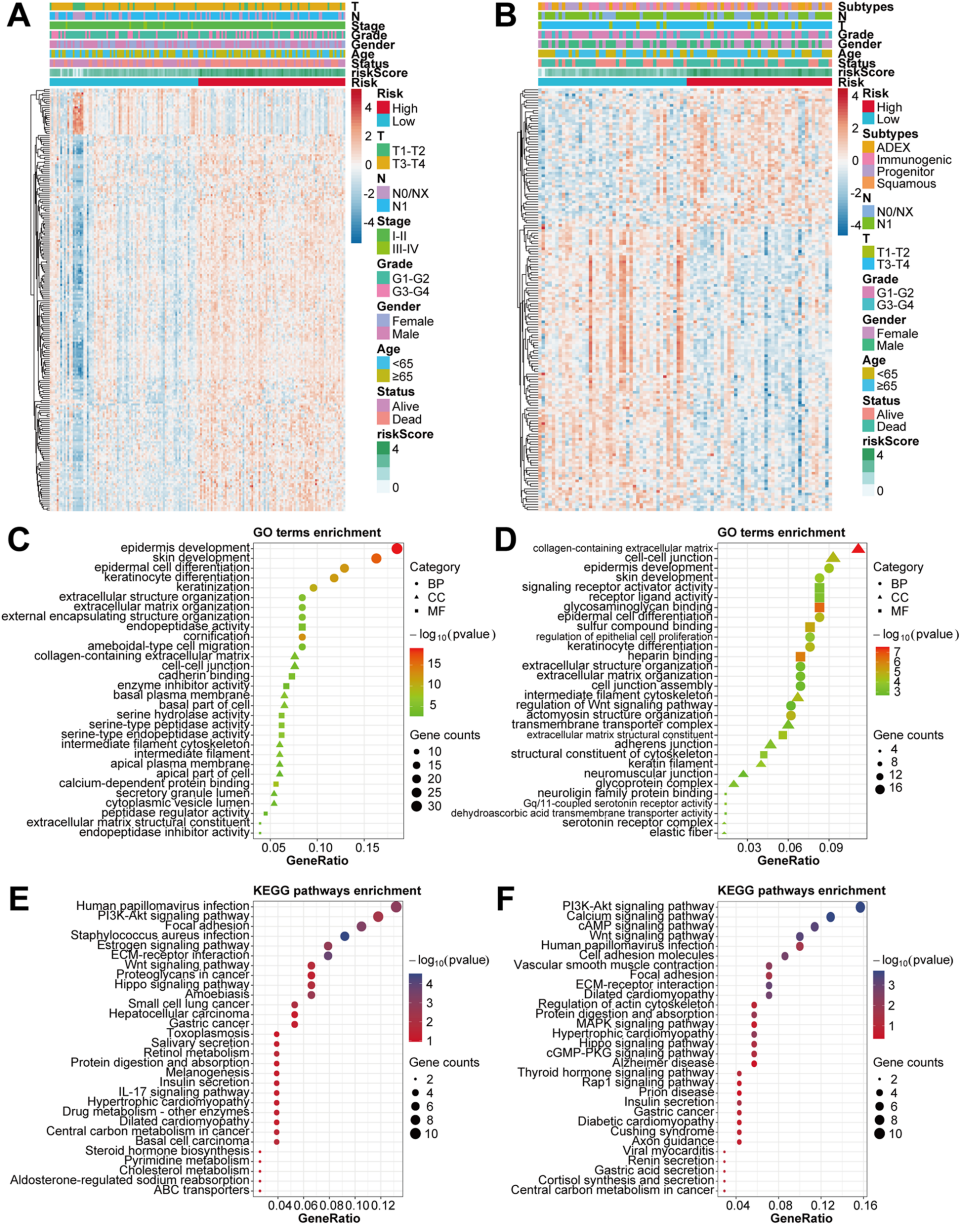
Differential gene expression analysis, GO and KEGG enrichment analyses between two risk groups. **A **and **B** Heatmap of the DEGs between the high-risk and low-risk groups in TCGA (A) and ICGC (B) cohorts. **C − F** Representative terms of GO enrichment analysis and representative pathways of KEGG enrichment analysis between the high-risk and low-risk groups in TCGA (C and E) and ICGC (D and F) cohorts

**Fig. 5:**
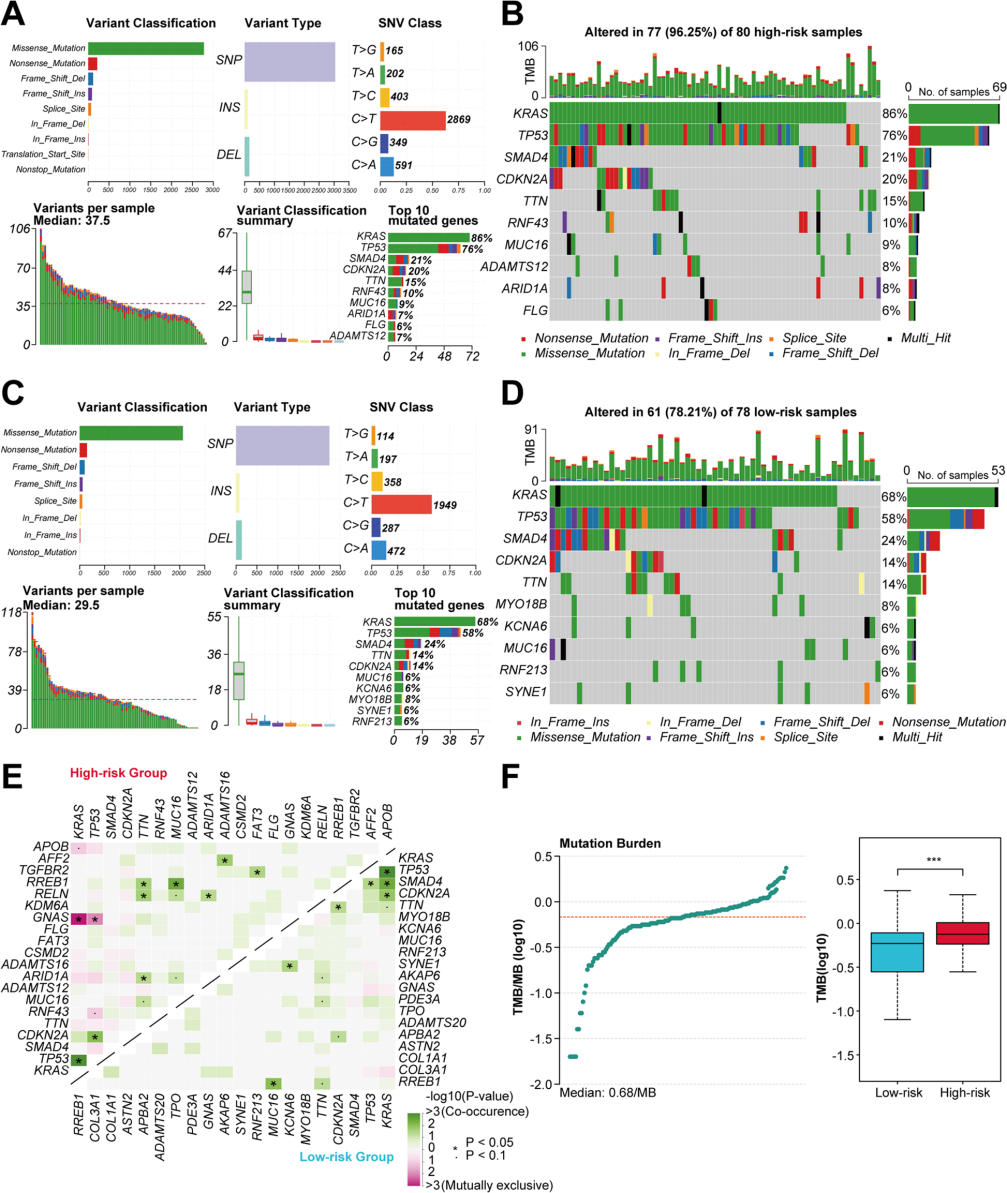
Somatic mutation profiles between two risk groups in TCGA cohort. **A − D** MAF-summary plots and waterfall charts of somatic mutations in the high-risk group (A and B) and low-risk group (**C** and **D**). The top 10 mutated genes were shown **E** Correlation heatmaps of co-occurrence and mutually exclusive mutations in the high-risk and low-risk groups. **F** Distribution of TMB (left) and comparison between two risk groups (right). ***, *P* < 0.001.

To further explore the biological functions and pathways associated with the established prognostic model, we first analyzed the DEGs between the high-risk and low-risk groups (Fig. 4A and B). A total of 212 DEGs were identified in TCGA cohort, including 189 upregulated and 23 downregulated genes. Moreover, in ICGC cohort, 162 DEGs were identified, including 52 upregulated and 110 downregulated genes. Notably, *KRT7* was one of the top 10 upregulated genes sorting by adjust *P* values in both TCGA and ICGC cohorts (Fig. S3A and B). The GO analysis showed that DEGs were enriched in several cell differentiation and tumor metastasis-related processes, such as epidermal cell differentiation, extracellular matrix organization, ameboidal-type cell migration, intermediate filament cytoskeleton, and cell–cell junction (Fig. 4C and D). The KEGG pathway analysis demonstrated that DEGs were mainly enriched in several pathways associated with invasiveness and metastasis of cancer, such as PI3K − Akt signaling pathway, Wnt signaling pathway, Hippo signaling pathway, Focal adhesion, ECM − receptor interaction, and Regulation of actin cytoskeleton (Fig. 4E and F). To clarify whether the risk score was associated with the mutational landscapes of PC patients, we compared the somatic mutation profiles between the high-risk and low-risk groups in TCGA cohort (Fig. 5A − D). Notably, the mutation frequency in the high-risk group was 96.25%, while 78.21% in the low-risk group, indicating that the mutation frequency increased with the risk score. Moreover, *KRAS* and *TP53* were the top two genes with the highest mutation frequencies in both subgroups. We also found that more co-occurrence and mutually exclusive mutations were observed in the high-risk group when compared with the low-risk group (Fig. 5E). In addition, patients with higher risk scores demonstrated higher TMB levels (*P* < 0.001; Fig. 5F). We further conducted the same analyses in ICGC cohort and similar results were verified (Fig. S4).

### Analyses of immune features between two risk groups

**Fig. 6:**
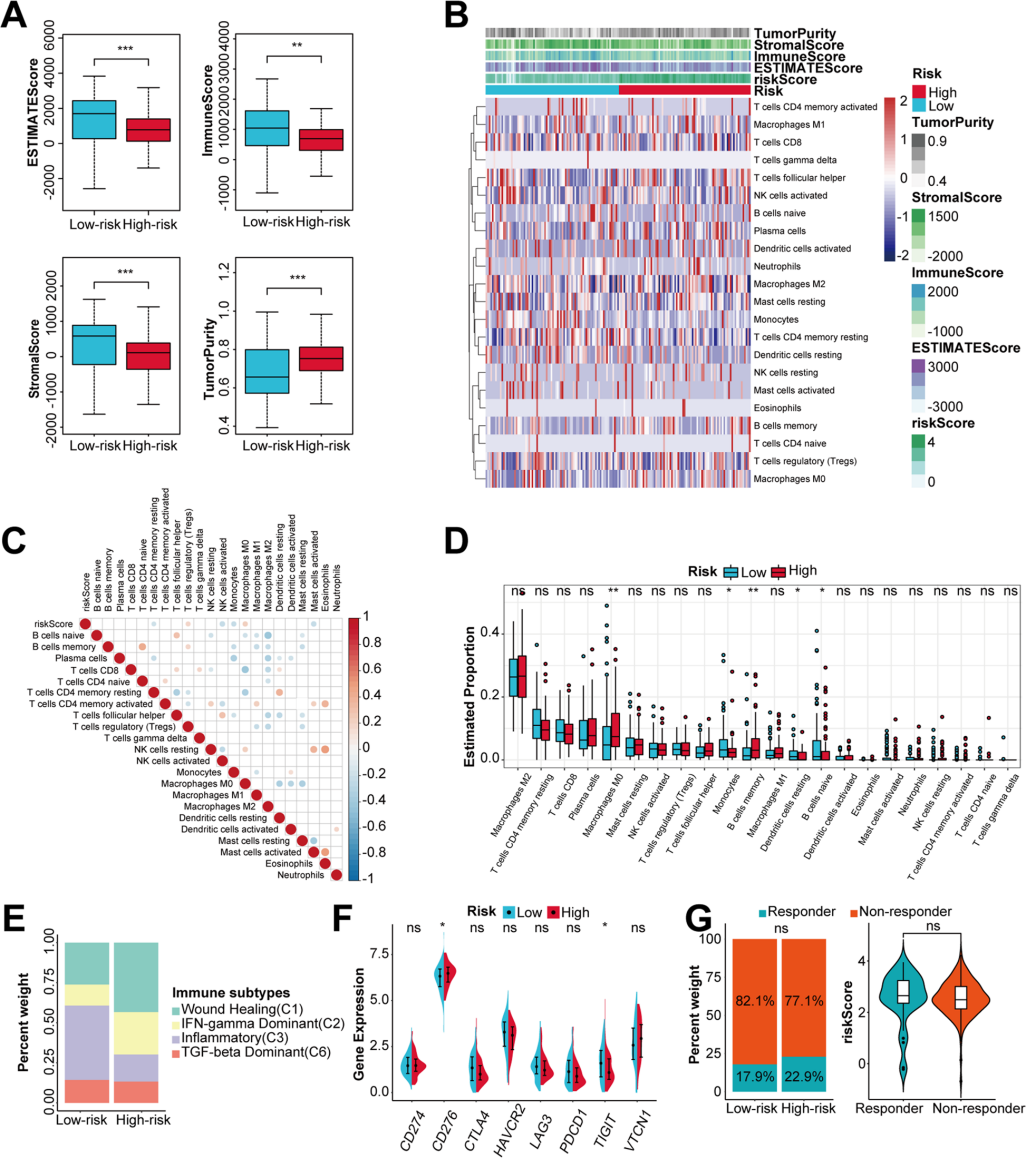
Estimation of immune cell infiltration and prediction of ICB responses in TCGA cohort. **A** Comparison of estimate score, immune score, stromal score and tumor purity between two risk groups. **B** Heatmap displaying the infiltrating abundances of 22 types immune cells. **C** Correlation heatmap of 22 types immune cells and the risk score. **D** Comparison of CIBERSORT scores of 22 types immune cells between two risk groups. **E** Proportions of four immune subtypes in two risk groups. **F** The expression levels of eight immune checkpoints between two risk groups. **G** Comparison of ICB response rates between two risk groups and the risk score between responders and non-responders. ns, not significant; *, *P* < 0.05, **, *P* < 0.01, ***, *P* < 0.001

PC harbors a highly heterogeneous TME. To further investigate whether the differences in prognosis between two risk groups were associated with immune cell infiltration, we used ESTIMATE and CIBERSORT algorithms to explore the immune infiltration levels in TCGA and ICGC cohorts, which highlighted that the high-risk group was characterized by a lower estimate score, stromal score and immune score, but a higher tumor purity (Fig. 6A, Fig. S5A). The composition and correlation of tumor-infiltrating immune cells showed that the risk signature was positively correlated with T cells regulatory (Tregs) and Macrophages M0, and negatively correlated with T cells CD4 memory resting, natural killer (NK) cells activated, Monocytes, Mast cells activated (Fig. 6B and C) and Macrophages M2 (Fig. S5B and C). Patients in the high-risk group exhibited significantly higher infiltrating levels of B cells memory, Macrophages M0 (*P* < 0.01, Fig. 6D) and Mast cells activated (*P* < 0.05, Fig. S5D), while lower infiltrating levels of B cells naive, Dendritic cells resting, Monocytes (*P* < 0.05, Fig. 6D) and T cells CD8 (*P* < 0.01, Fig. S5D). Subsequently, the classification of immune subtypes showed that four subtypes and five subtypes were identified in TCGA cohort and ICGC cohort, respectively (Fig. 6E, Fig. S5E). Patients in the high-risk group had more C1 (wound healing) and C2 (IFN-γ dominant) subtypes, and less C3 (inflammatory) subtype, suggesting an unfavorable prognosis. Emerging evidence has shown that immune features of TME are associated with the immune checkpoint blockade (ICB) therapeutic responses. The expression levels of eight representative immune checkpoints including *CD274* (PD-L1), *CD276* (B7-H3), *CTLA4* (CTLA-4), *HAVCR2* (TIM3), *LAG3* (LAG-3), *PDCD1* (PD-1), *TIGIT* (VSIG9) and *VTCN1* (B7-H4) were compared between two risk groups. We found that *CD276* (Fig. 6F), *CD274* and *VTCN1* (Fig. S5F) were upregulated in the high-risk group, while *TIGIT* was downregulated (Fig. 6F). Besides, the results of ICB responses prediction demonstrated that the response rates were higher in the high-risk group, and responders had higher risk scores (Fig. 6G, Fig. S5G). Although the rates of response and the risk scores between two groups were not statistically significant in TCGA cohort, an increasing tendency was observed in the high-risk group and responders, respectively. These findings indicated that patients with higher risk scores might be in an immune-suppressive status.

### Correlation between CIC-related risk score and other molecular subtypes of PC

Previous studies have identified and validated biologically and clinically relevant molecular subtypes of PDAC based on gene expression profiles [29–31]. In this study, we analyzed the correlation between CIC-related risk score and two established PDAC subtypes defined by Bailey et al. and Moffitt et al. Our results demonstrated that CIC-related risk score was comparable among Bailey’s subtypes (Immunogenic, ADEX, Pancreatic progenitor and Squamous subtypes) (Fig. S6A). But Squamous subtype that had the worst prognosis in Bailey’s cohort showed the highest proportion of patients who were divided into the CIC-related high-risk group (68% of patients with this subtype) (Fig. S6B). The combination of Bailey’s subtypes and CIC-related risk score in the Kaplan–Meier survival analysis also exhibited that patients with Squamous subtype plus CIC-related high-risk had the worst prognosis (Fig. S6C). In Moffitt’s subtypes (Classical and Basal-like subtypes), Basal-like subtype showed higher CIC-related risk scores and 71% patients with this subtype were divided into the CIC-related high-risk group (Fig. S6D-E). The combination of Moffitt’s subtypes and CIC-related risk score stratified PDAC patients into four group and patients with Basal-like subtype demonstrated the worse prognosis (Fig. S6F).

### Higher expression of KRT7 correlates with an unfavorable prognosis in PC

**Fig. 7:**
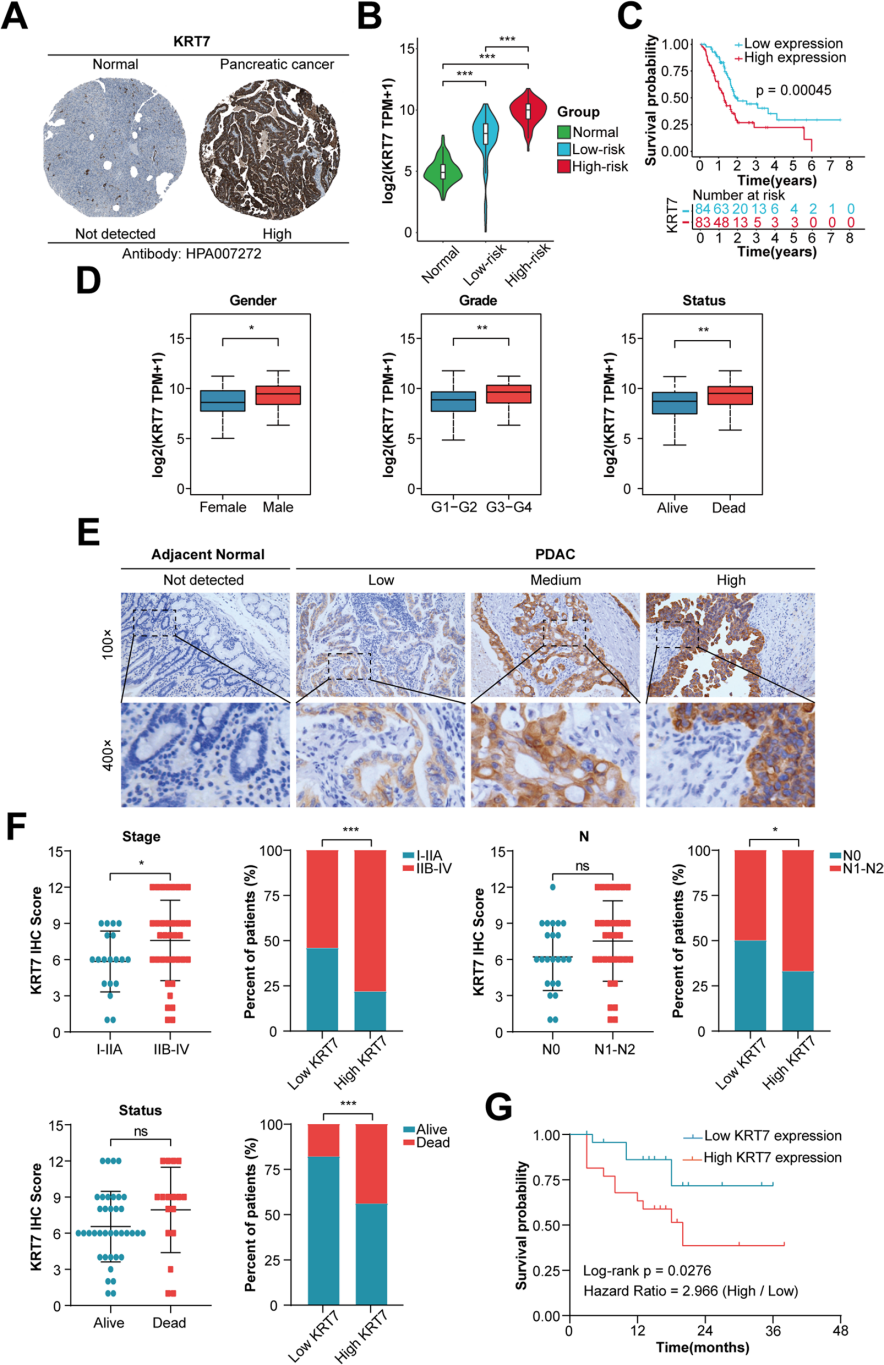
Validation of KRT7 high expression in PC and its association with poor prognosis in TCGA and PUMCH cohorts. **A** Representative images of IHC staining of KRT7 in normal and PC tissues from HPA database. **B** Comparison of *KRT7* expression between normal samples (GTEx dataset) and tumor samples (TCGA dataset). TCGA patients were stratified into the low-risk and high-risk groups based on the risk score of individuals. **C** The Kaplan–Meier survival analysis based on *KRT7* expression in TCGA cohort. **D** The comparison of *KRT7* expression between gender, grade and survival status subgroups in TCGA cohort. **E** Representative images of IHC staining of KRT7 in PUMCH cohort (*n* = 55). **F** The comparison of KRT7 IHC scores between AJCC stage, N stage and survival status subgroups, and proportions of these characteristics between KRT7 low- and high-expression subgroups in PUMCH cohort. Low expression, IHC scores 1 − 6; high expression, IHC scores 8 − 12. Patients were stratified into the low- and high-expression groups based on the median IHC score (median value = 6). **G** The Kaplan–Meier survival analysis of 55 PDAC patients from PUMCH cohort. ns, not significant; *, *P* < 0.05, **, *P* < 0.01 and ***, *P* < 0.001

As shown in the results of multivariate Cox regression and correlation analysis, *KRT7* was the most important risk gene in the established prognostic model for significantly predicting prognosis of PC patients and positively correlated with other three genes expression (Fig. 1G and H). We investigated the protein and mRNA expression levels of KRT7 by HPA database, GTEx and TCGA datasets. We found that the expression of KRT7 in PC tissue was significantly higher than normal pancreas tissue and the expression level increased with the risk score elevation (Fig. 7A and B). Meanwhile, the survival analysis showed that, based on the median of *KRT7* expression level, patients with a higher expression of *KRT7* had a shorter OS (Fig. 7C). Moreover, the expression of *KRT7* between differently clinicopathological subgroups were further compared. Notably, the expression of *KRT7* was significantly increased in male, higher grade and death (Fig. 7D, Fig. S7A). Then, we performed IHC staining of KRT7 in 55 PDAC samples from our own PUMCH cohort to further validate that KRT7 high expression was associated with unfavorable prognosis in PC (Table 2, Fig. 7E). According to the median of IHC score, we next divided patients into low and high KRT7 subgroups. Combined with KRT7 IHC scores and differently clinicopathological characteristics, patients of high KRT7 group showed higher proportions of IIB-IV stage, lymphatic metastasis and death (Fig. 7F, Fig. S7B). Consistent with the result of survival analysis in TCGA cohort, high KRT7 group demonstrated poorer prognosis (*P* = 0.0276, Fig. 7G).

**Table2:** Clinical characteristics of pancreatic cancer patients in the PUMCH cohort

Variables	Total	KRT7 Low expression	KRT7 High expression
	*n* = 55 (%)	*n* = 28 (%)	*n* = 27 (%)
**Age**			
< 65	28 (50.9)	16 (57.1)	12 (44.4)
≥ 65	27 (49.1)	12 (42.9)	15 (55.6)
**Gender**			
Female	25 (45.5)	14 (50.0)	11 (40.7)
Male	30 (54.5)	14 (50.0)	16 (59.3)
**Tumor grade**			
Well	4 (7.3)	2 (7.1)	2 (7.4)
Moderately	32 (58.2)	16 (57.1)	16 (59.3)
Poorly	19 (34.5)	10 (35.8)	9 (33.3)
**Stage**			
I − IIA	19 (34.5)	13 (46.4)	6 (22.2)
IIB − IV	36 (65.5)	15 (53.6)	21 (77.8)
**T**			
T1 − T2	35 (63.6)	17 (60.7)	18 (66.7)
T3 − T4	20 (36.4)	11 (39.3)	9 (33.3)
**N**			
N0	23 (41.8)	14 (50.0)	9 (33.3)
N1 − N2	32 (58.2)	14 (50.0)	18 (66.7)
**M**			
M0	51 (92.7)	27 (96.4)	24 (88.9)
M1	4 (7.3)	1 (3.6)	3 (11.1)
**Status**			
Alive	38 (69.1)	23 (82.1)	15 (55.6)
Dead	17 (30.9)	5 (17.9)	12 (44.4)
**OS time (months)**			
Median (range)	15 (3 − 38)	15 (3 − 36)	13 (3 − 38)
**KRT7 IHC score**			
Median (range)	6 (1 − 12)	6 (1 − 6)	9 (8 − 12)

*PUMCH* Peking Union Medical College Hospital, *OS* Overall survival,

*IHC* Immunohistochemistry

### High expression of KRT7 is associated with suppressive immune microenvironment in PC

**Fig. 8:**
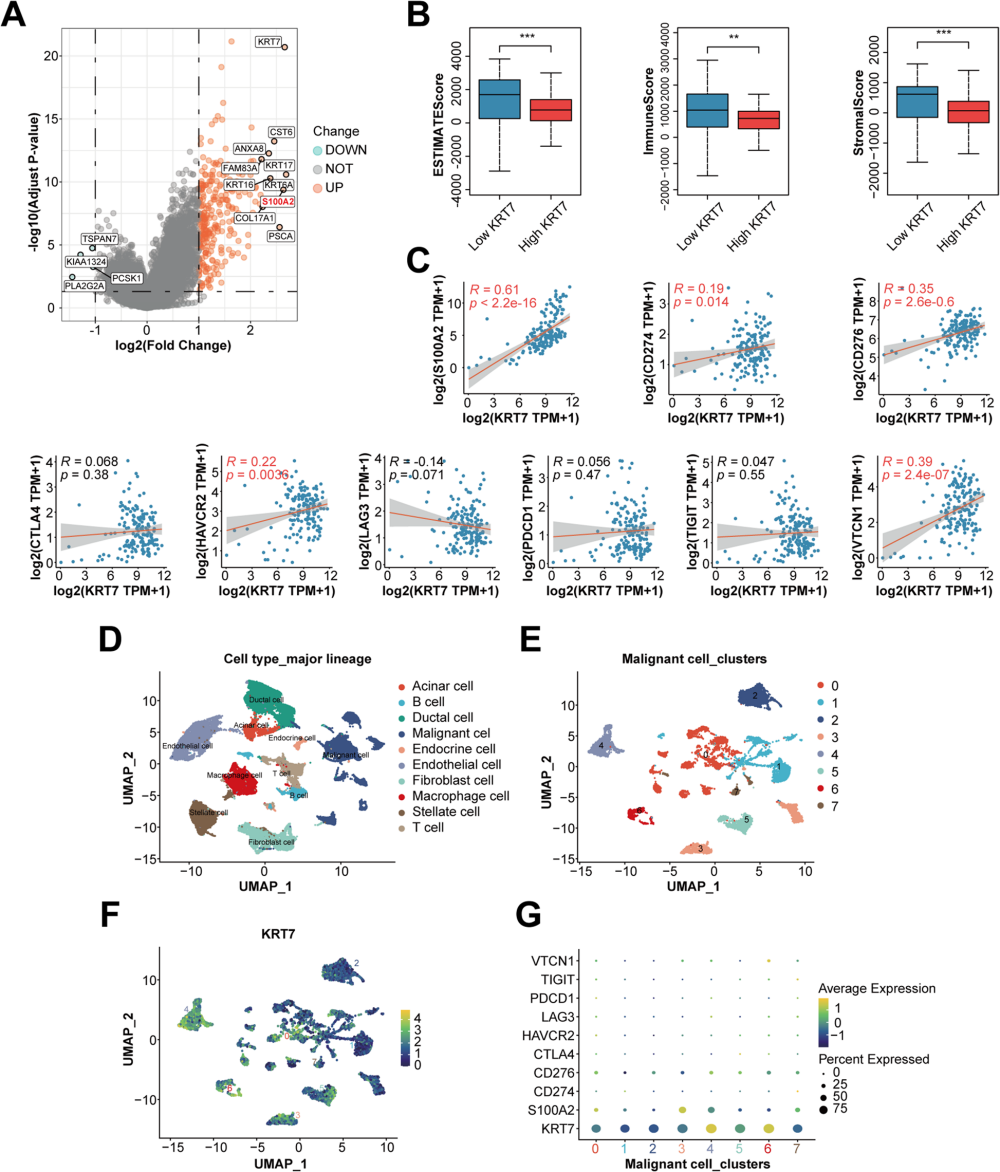
Identification of the correlation between *KRT7* high expression and immune-suppressive status based on integrated analysis of bulk-seq and scRNA-seq. **A** Volcano plot of the DEGs between the high and low *KRT7* expression groups in TCGA cohort. Patients were stratified into the high and low groups according to the median of *KRT7* expression. **B** Comparison of estimate score, immune score and stromal score between the high and low *KRT7* expression groups in TCGA cohort. **C** Correlation analyses between the expression of *KRT7* and immune-related genes, including eight immune checkpoints in TCGA cohort. **D and E** The UMAP plots of diverse cell types in PDAC tissues colored by major cell lineage (**D**) and eight malignant clusters colored by cluster (E). **F** Relative expression of *KRT7* among eight malignant clusters visualized by UMAP projection. **G** Relative expression of *KRT7* and immune-related genes in each malignant clusters visualized by dot plot. **, *P* < 0.01 and ***, *P* < 0.001

To investigate the potential mechanism of KRT7 expression worsening the prognosis of PC, differential gene expression analysis was performed between *KRT7* high- and low-expression group in TCGA cohort. Interestingly, *S100A2* (S100 Calcium Binding Protein A2), as a prognostic biomarker involved in immune infiltration and immunotherapy response in PC, was one of the top 10 upregulated genes (Fig. 8A). Therefore, we applied the ESTIMATE algorithm to estimate levels of infiltrating stromal and immune cells, where the results showed that *KRT7* high-expression group has a significantly lower estimate score, stromal score, and immune score (Fig. 8B). In addition, we discovered that the expression of *KRT7* was positively correlated with the expression of *S100A2* and representative immune checkpoints, including *CD274*, *CD276*, *HAVCR2*, and *VTCN1* (Fig. 8C). Given the extensive degree of intra-tumoral heterogeneity in PC, we further examined the expression of *KRT7* among different cell clusters in TME on single-cell level. We identified 10 main cell types including malignant, ductal, acinar, endocrine, endothelial, fibroblast, stellate, macrophage, T and B cells (Fig. 8D). We then analyzed malignant cells and further divided them into 8 subgroups (cluster 0 − 7, Fig. 8E). By comparing gene expression levels among different clusters, we found that *KRT7* was highly expressed in cluster 4, 5 and 6, accompanying with a higher expression of *S100A2* or *CD276* (Fig. 8F and G). We also analyzed the gene expression features and correlations with prognosis of *AURKA*, *CDKN2A* and *RARB*, but none of them was as significant as *KRT7* (Fig. S8). These findings suggest that the expression of *KRT7* is correlated with immunosuppression in PC microenvironment and malignant cells with higher *KRT7* expression may have greater resistance to anti-tumor immunity.

### Expression of KRT7 is associated with cic formation, cell cluster, cell proliferation, migration, and invasion of PC cell lines

**Fig. 9:**
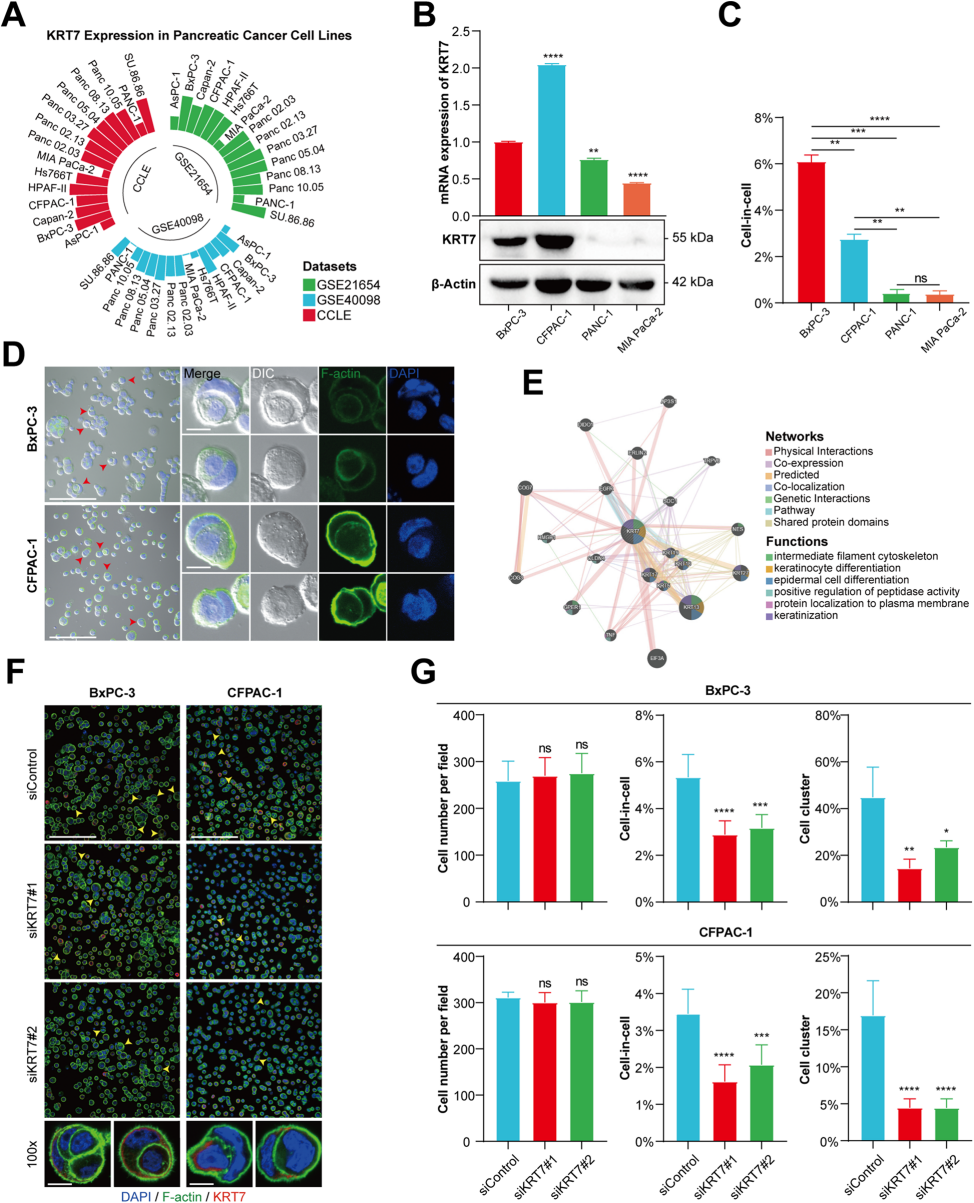
Identification of the correlation between *KRT7* expression and CIC formation in PC cell lines. **A** *KRT7* expression levels among 15 PC cell lines in three independent datasets. **B** The expression levels of *KRT7* mRNA and protein in BxPC-3, CFPAC-1, PANC-1 and MIA PaCa-2 cell lines. **C** The frequencies of CIC formation in four cell lines. **D** Representative immunofluorescent images of typical CIC structures for BxPC-3 and CFPAC-1 cells. Red arrows indicate CIC structures. Scale bar: 100 μm (left) and 10 μm (right). **E** Related networks and functions of *KRT7* predicted by GeneMANIA website. **F** Representative immunofluorescent images of typical CIC structures for BxPC-3 and CFPAC-1 cells transfected with siRNA of *KRT7* or negative control siRNA. Yellow arrows indicate CIC structures. Scale bar: 100 μm (top) and 10 μm (bottom). **G** The frequencies of CIC formation and cell cluster for KRT7-knockdown BxPC-3 and CFPAC-1 cells. Data represent means ± SD from three independent experiments. ns, not significant; *, *P* < 0.05; **, *P* < 0.01; ***, *P* < 0.001; ****, *P* < 0.0001

**Fig. 10:**
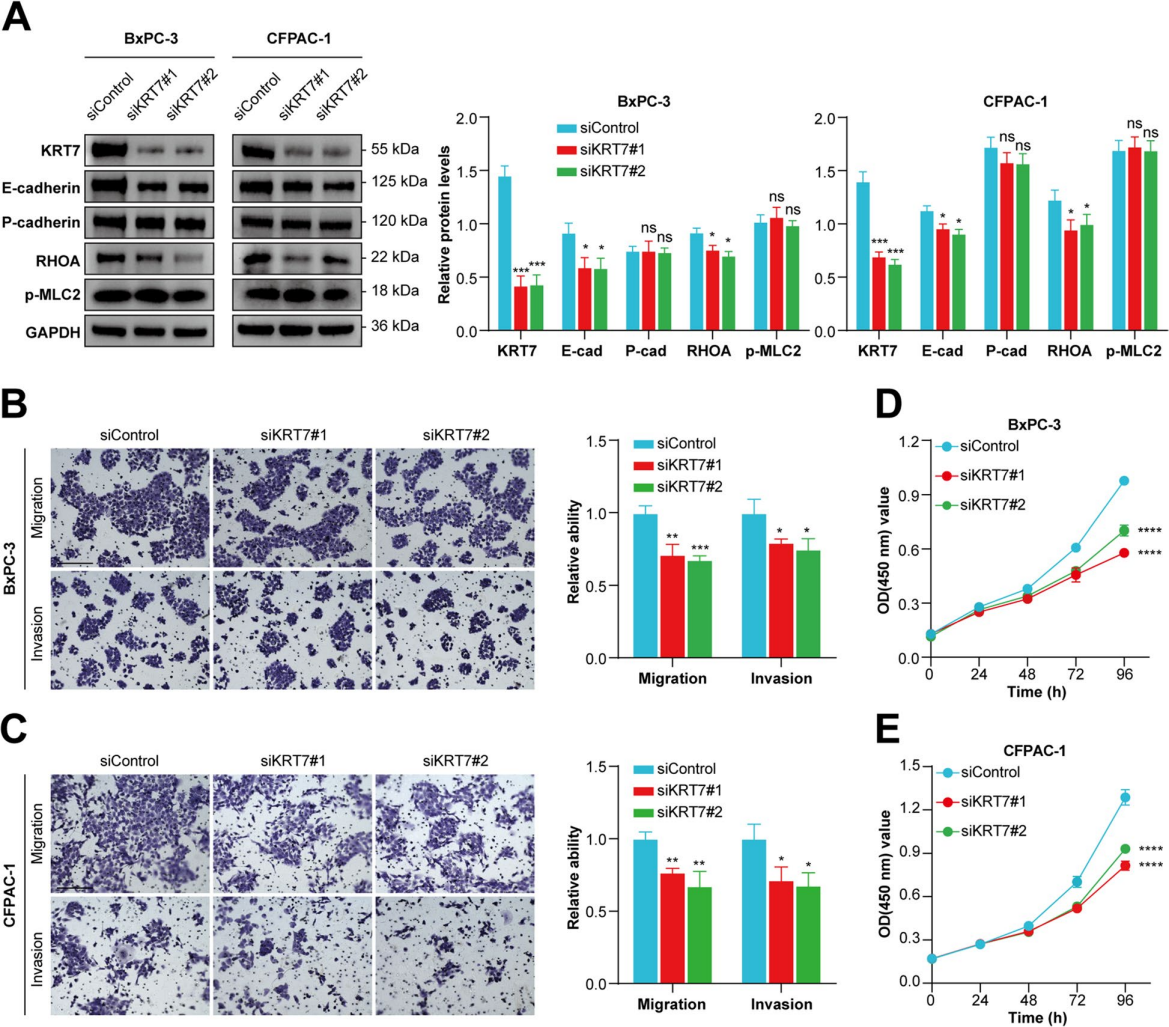
Silencing *KRT7* expression downregulated the expression of E-cadherin and RHOA, and inhibited the cell proliferation, migrative and invasive abilities in both BxPC-3 and CFPAC-1 cell lines. **A** Western blot images and densitometric quantification of CIC-related protein expression levels in *KRT7*-knockdown BxPC-3 and CFPAC-1 cells. Relative expression levels were normalized to GAPDH loading control. **B **and **C** Effects of silencing *KRT7* expression on migration and invasion of BxPC-3 and CFPAC-1 cell lines. Scale bar: 200 μm. **D and E** Effects of silencing *KRT7* expression on cell proliferation of BxPC-3 and CFPAC-1 cell lines. Data represent means ± SD from three independent experiments. ns, not significant; *, *P* < 0.05; **, *P* < 0.01; ***, *P* < 0.001; ****, *P* < 0.0001

To clarify whether *KRT7* expression is associated with CIC formation, we first compared the expression levels of *KRT7* among different PC cell lines in three independent datasets (Fig. 9A). Four cell lines with relatively higher and lower expression of *KRT7* at both mRNA and protein levels were selected respectively for CIC formation assay (Fig. 9B). We found that BxPC-3 and CFPAC-1 cell lines with relatively higher expression levels of *KRT7* showed higher frequencies of CIC formation than PANC-1 and MIA PaCa-2 with relatively lower expression levels of *KRT7* (Fig. 9C and D). The related networks and functions of *KRT7* mainly involve in cytoskeleton remodeling, cell differentiation, and protein localization-related functions (Fig. 9E). Next, we knocked down the expression of *KRT7* in BxPC-3 and CFPAC-1 cell lines. Silencing the expression of *KRT7* by siRNA significantly inhibited CIC formation and reduced cell clusters in both cell lines (Fig. 9F and G). We also found that *KRT7* knockdown decreased the expression of E-cadherin and RHOA, which may be responsible for the decline of CIC frequencies (Fig. 10A). Furthermore, silencing the expression of *KRT7* also suppressed the proliferation, migration, and invasion of both cell lines (Fig. 10B − E).

## Discussion

The global burden of PC has increased continuously over the past few decades, and as the leading cause of cancer-related death worldwide, its 5-year survival rate approached 10% for the first time in 2020 [1]. Due to the lack of an effective screening for PC at an early stage, most patients are diagnosed at locally advanced or metastatic stage. Even after standardized treatment, including surgery and adjuvant chemotherapy, patients still have to face the great risk of recurrence and death [3]. Therefore, reliable biomarkers for early detection screening and predicting OS of PC patients are urgently needed.

Acquiring necessary nutrients from a frequently nutrient-poor environment and utilizing these nutrients to maintain rapid proliferation and progression is a common feature of cancer cell metabolism [32]. Beyond scavenging nutrients from extracellular microenvironment, cancer cells also engulf and digest whole living cells via two mainly CIC processes, “cell cannibalism” and “entosis”, for nutrient recovery [33]. Previous studies have demonstrated that cannibalistic activity is a hallmark of cancer, conferring metabolic advantages on cancer cells under energy stress [8, 15]. Meanwhile, cancer cells with higher aggressiveness can become the “winner” subpopulation eliminating their less competitive neighbors, which promotes the fittest clones expanding within heterogeneous cancer cell populations [16]. CIC structures are prevalent in PC tissues and homotypic CIC (cancer cells internalized other cancer cells) constitutes the main subtype of overall CIC structures in PC [7, 34]. Considering that CIC phenomena in cancer represent a highly aggressive behavior, indicating that CIC-mediated cell competition, by selecting the best competitive clones, may be a potential mechanism to promote PC progression. However, no previous studies investigated the relationship between CIC-related genes and PC patient’s prognosis through comprehensive bioinformatics analysis.

In present study, we developed a risk scoring model based on four CIC-related genes (*KRT7*, *AURKA*, *CDKN2A* and *RARB*) in TCGA cohort, and further performed external validation for its robustness. According to the values of hazard ratio, *KRT7* and *AURKA* were considered as the risk genes, while *CDKN2A* and *RARB* were considered to be protective. KRT7 belongs to type II cytokeratin involving in cytoskeleton remodeling, epithelial intermediate filaments formation, and motility enhancement of cells [35]. Previous studies have indicated that KRT7 is overexpressed in many cancers, including ovarian, gastric, colorectal, and pancreatic cancer, which can facilitate the migration and invasion of cancer cells [36–39]. AURKA is a regulator kinase of cell cycle involved in microtubule formation, spindle pole stabilization during chromosome segregation, and functionally contributes to tumorigenesis and progression [40]. AURKA-mediated phosphorylation is necessary for CIC-related processes, which promotes entosis in breast cancer cells through the regulation of microtubule plus-end dynamics and cell rigidity [41]. CDKN2A, a well-known tumor suppressor, can induce cell cycle arrest in G1 and G2 phases by inhibiting the binding of CDK4 or CDK6 with cyclin D1 and initiating p53-dependent cell cycle arrest. CDKN2A inactivation is present in the majority of PC patients, increasing cellular fitness and proliferation, and promotes homotypic CIC formation in breast cancer cells [28, 42]. RARB, by binding retinoic acid (biologically active vitamin A), can limit cell proliferation and abnormality to inhibit tumorigenesis. Various studies have suggested that cancer cells elevate the expression levels of RARB promoter methylation and result in functional silencing [43, 44].

Based on the risk score of individuals, patients were divided into the low- and high-risk groups. Our results showed that PC patients in the high-risk group had significantly poorer OS than those in the low-risk group, and the risk score was an independent prognostic factor with a credible efficacy confirmed by ROC analysis and nomogram model. To further explore the associations between established signature and differences of prognosis observed, biological functions, mutation profiles and immune features between two risk groups were compared in TCGA and ICGC cohorts. Functional enrichment analyses showed that several cancer-related terms and pathways were enriched, such as extracellular matrix organization, cell–cell junction, ECM-receptor interaction and PI3K-Akt signaling pathway, suggesting that patients in the high-risk group may be at higher degree of cancer-related pathways activation and immunosuppressive status [45]. Furthermore, a higher proportion of patients with *KRAS* and *TP53* somatic mutations were detected in the high-risk group, two well-known drivers of PC, increasing the risk of cancer in individuals. TMB, a numeric index, reflects cancer mutation quantity. Thus, a higher TMB results in more tumor neoantigens, which increases chances for immunotherapy and is clinically associated with better ICB responses [46]. In this study, patients in the high-risk group had significantly higher TMB, indicating that these patients may benefit from ICB therapy. Previous studies have shown that the immune microenvironment plays a pivotal role in the progression of PC [45, 47, 48]. However, the roles of CIC-related genes for PC immune microenvironment are still unclear. In our results, higher infiltration levels of M0 macrophages and lower infiltration levels of CD8^+^ T cells were observed in the high-risk group, suggesting the presence of an immunosuppressive microenvironment [49]. Besides, it has been reported that tumor-infiltrating B cells contribute to a better response to ICB therapy, and tumors of responders showed a higher infiltrating level of memory B cells, while lower infiltrating level of naïve B cells than tumors of non-responders [50]. This finding may be the reason for our results that there are a significantly higher frequency of memory B cells and a significantly lower frequency of naïve B cells in the high-risk group. A previous study, by analyzing TCGA data, has identified six immune subtypes spanning cancer tissue types and molecular subtypes − wound healing (C1), IFN-γ dominant (C2), inflammatory (C3), lymphocyte depleted (C4), immunologically quiet (C5), TGF-β dominant (C6). C3 subtype has the best prognosis, while C1 and C2 subtypes present less favorable outcomes [51]. In our results, nearly half of patients in the low-risk group were C3 subtype, but most patients in the high-risk group were C1 and C2 subtypes, which was consistent with the association of immune subtypes and prognosis. ICB therapy is one of the most successful anti-cancer immunotherapies [52]. However, low response rates limit PC patients to benefit from ICB therapy [3]. We analyzed the expression of eight representative immune checkpoints between two risk groups and predicted ICB responses of individuals. As shown in our results, the high-risk group exhibited higher expression of *CD274*, *CD276* and *VTCN1* than the low-risk group. *CD274* encodes PD-L1 protein, the immune inhibitory ligand of PD-1 death receptor, that is observed in various types of tumors, and serves as an immune suppressor by blocking T cells activation and cytokine production [52]. *CD276* is widely expressed on a range of solid tumors, and plays a dual role in anti-tumor immunity, as a co-stimulatory regulator enhancing the activity of T cells, or as a co-inhibitory regulator inhibiting T cells and NK cells functions [53].*VTCN1* is mainly expressed on tumor cells and tumor-associated macrophages, which promotes immune escape by inhibiting the proliferation of T cells and enhancing the function of regulatory T cells [54]. *TIGIT*, as the only downregulated immune checkpoint in the high-risk group, is primarily expressed on T cells and NK cells, which can inhibit anti-tumor immunity by impairing T cell functions, preventing NK cell-mediated lysis, and enhancing the suppressive activity of regulatory T cells [55]. Therefore, these findings may explain why a higher frequency ICB response rate was observed in the high-risk group.

As the most important risk gene in our prognostic model, we performed IHC scoring of KRT7 on PUMCH cohort to further validate the correlation of KRT7 expression with an unfavorable prognosis of PC. According to the comprehensive analysis of IHC scores and clinicopathological characteristics, high expression of KRT7 was significantly associated with higher stage, lymphatic metastasis, and shorter OS time. High intra-tumoral heterogeneity is the main obstacle in fulfilling an effective PC treatment [56]. The results of single-cell transcriptome analysis for PC have found that malignant cells contain distinct subpopulations, including those enriched for either proliferative or migrative features [26, 47]. Meanwhile, this heterogeneity is also found in established cancer cell line such as neuroblastoma, melanoma and breast carcinoma cell lines [57, 58]. Heterogeneous tumor populations can be divided into separate clusters with differently mechanical deformability [58]. Our results showed that the expression levels of *KRT7* were varied among different malignant clusters and established cell lines, which may confer different degrees of the deformability and motility to tumor cells. Moreover, silencing of *KRT7* significantly diminished the CIC formation, cell cluster, cell proliferative, migrative and invasive abilities. Considering that softer tumor cells preferentially internalize stiffer neighboring cells in CIC processes, we speculate that CIC structures observed in PC may be a positive selection to promote the survival of clones with metabolic advantages and higher deformability, which promotes the invasion and metastasis of PC cells (unpublished data) [16, 59].

To the best of our knowledge, this study is the first attempt to construct a prognostic model based on CIC-related genes and has shown a favorable efficacy on survival prediction in PC patients. However, our study has several limitations. First, this study mainly collected retrospective data from public datasets, and thus prospective studies are needed to further validate our results. Meanwhile, an independent validation cohort with greater sample size and long-term follow-up is warranted in the future. Second, since there are only limited number of research delineated the dynamic CIC processes in PC, genes included in this study may perhaps not the core regulators of CIC in PC cells. Third, due to the nature of bioinformatics analysis, future studies are needed to further elucidate the molecular mechanisms and biological implications of CIC-related genes such as *KRT7* in PC progression.

In conclusion, our study developed a prognostic model for PC based on four CIC-related genes to screen patients at high risk and predict survival. KRT7 might be a contributor of the immunosuppressive microenvironment in PC and it has shown great potential as a novel prognostic marker. More studies are needed to reveal new insights about CIC phenomena in cancer progression, which may shed inspiring light on PC therapy.

## Supplementary Information

**Additional file 1.**

**Additional file 2.** 

## Data Availability

The public datasets could be downloaded at https://xenabrowser.net/ (cohort: TCGA TARGET GTEx and PACA-AU), https://portal.gdc.cancer.gov/ (project ID: TCGA-PAAD), http://dcc.icgc.org/ (project ID: PACA-AU), https://www.ncbi.nlm.nih.gov/geo/ (accession number: GSE21501, GSE62452, GSE71729, GSE21654 and GSE40098), https://sites.broadinstitute.org/ccle/ (File name: CCLE_expression) and https://ngdc.cncb.ac.cn/ (GSA number: CRA001160). Further inquiries can be directed to the corresponding author.

## Abbreviations

PC: Pancreatic cancer
CIC: Cell-in-cell
GTEx: Genotype-Tissue Expression
TCGA: The Cancer Genome Atlas
ICGC: International Cancer Genome Consortium
GEO: Gene Expression Omnibus
NGDC: National Genomics Data Center
LASSO: Least absolute shrinkage and selection operator
ROC: Receiver operating characteristic
GO: Gene Ontology
KEGG: Kyoto Encyclopedia of Genes and Genomes
DEGs: Differentially expressed genes
qRT-PCR: Quantitative real-time PCR
IHC: Immunohistochemistry
PDAC: Pancreatic ductal adenocarcinoma
RNA-seq: RNA sequencing
scRNA-seq: Single-cell RNA sequencing
UCSC: University of California Santa Cruz
TPM: Transcripts per million
CPM: Counts per million
CCLE: Cancer Cell Line Encyclopedia
FDR: False discovery rate
PCA: Principal component analysis
OS: Overall survival
HPA: Human Protein Atlas
TMB: Tumor mutation burden
TME: Tumor microenvironment
ATCC: American Type Culture Collection
STR: Short Tandem Repeat
IMDM: Iscove’s Modified Dulbecco Medium
DMEM: Dulbecco’s Modified Eagle Medium
BSA: Bovine serum albumin
siRNA: Short interference RNA
PUMCH: Peking Union Medical College Hospital
SD: Standard deviation
AUC: Area under curves
DCA: Decision curve analysis
Tregs: T cells regulatory
Natural killer cells: NK cells
ICB: Immune checkpoint blockade
KRT7: Keratin 7
AURKA: Aurora Kinase A
CDKN2A: Cyclin Dependent Kinase Inhibitor 2A
RARB: Retinoic Acid Receptor Beta
