# Supplementary material for: Construction of a novel model based on cell-in-cell-related genes and validation of KRT7 as a biomarker for predicting survival and immune microenvironment in pancreatic cancer
Source: BMC Cancer. 2022 Aug 16;22:894. doi: 10.1186/s12885-022-09983-6 (PMC9380297; doi:10.1186/s12885-022-09983-6)
Supplement: Supplementary file 1 — Additional file 1. [file 12885_2022_9983_MOESM1_ESM.pdf]

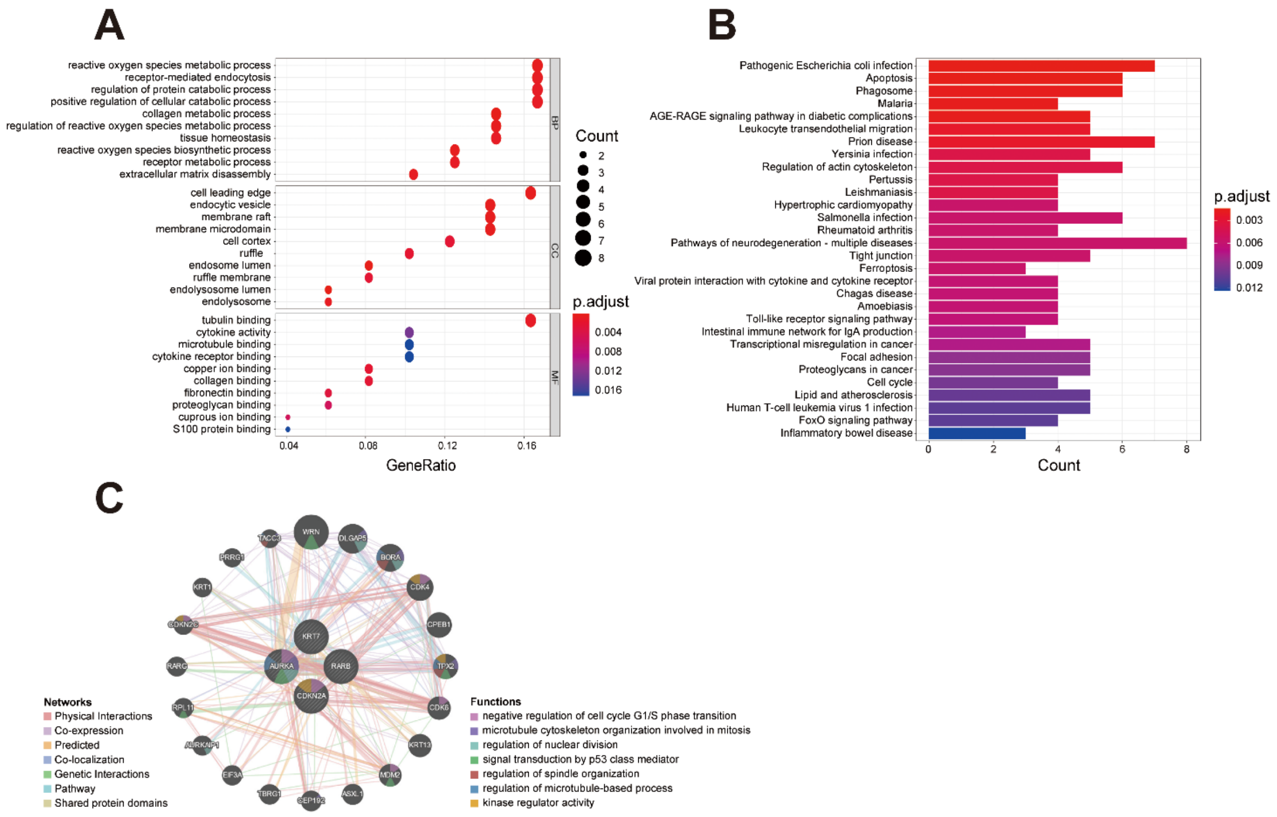

Fig.S1 Functional enrichment analyses of CIC-related DEGs and correlated networks prediction of four signature-related genes.

(A and B) GO (A) and KEGG (B) enrichment analyses based on 49 CIC-related DEGs. **(C)** Related networks and functions of four model genes predicted via GeneMANIA website.

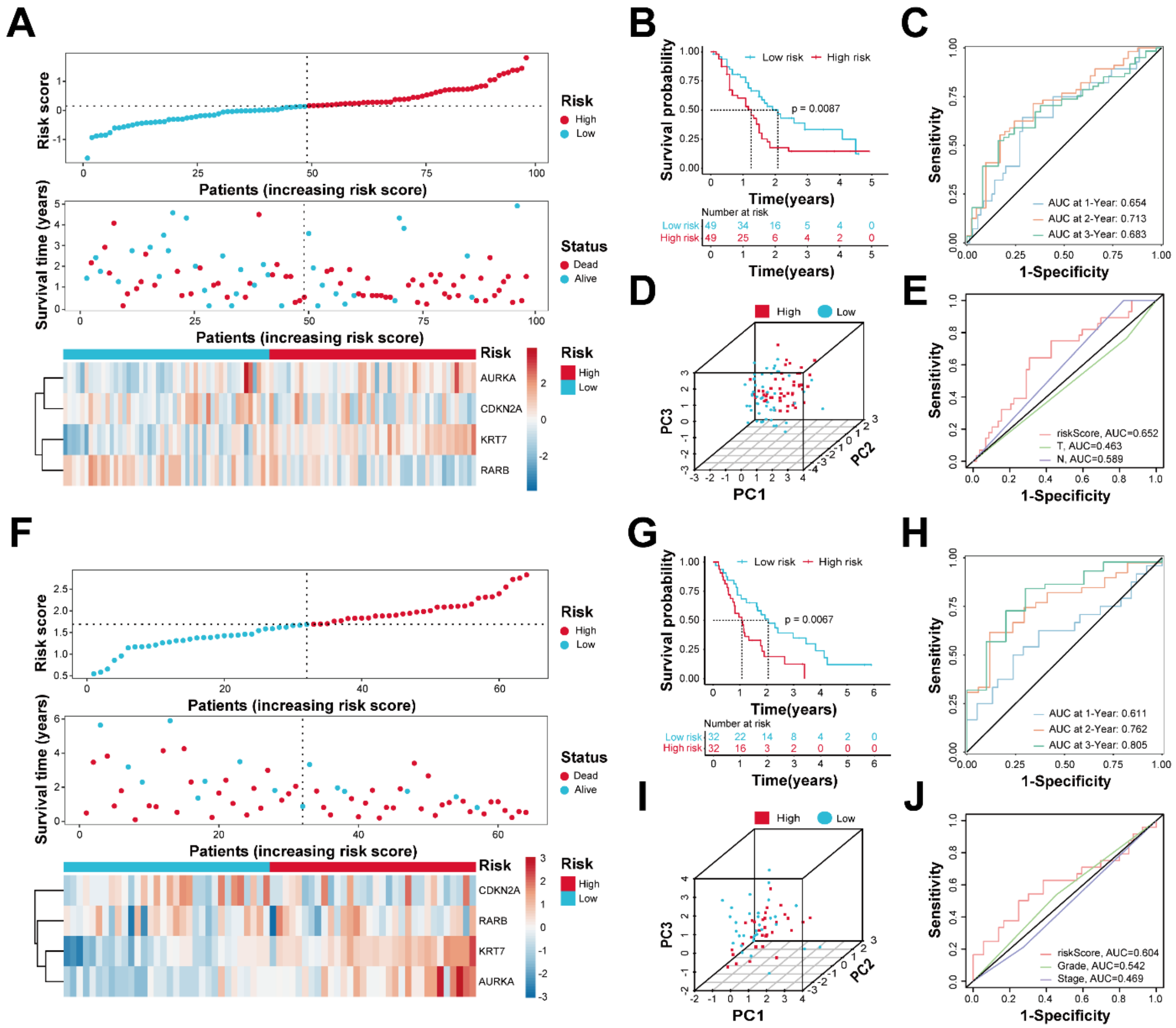

Fig.S2 Evaluation and validation of CIC-related prognostic signature in two GEO cohorts.

(A and F) Distribution of risk scores, OS status overview, and four genes expression in GSE21501 (A) and GSE62452 (F) cohorts. **(B and G)** Kaplan-Meier curves for the OS of patients between the high- and low-risk groups in GSE21501 (B) and GSE62452 (G) cohorts. **(C and H)** ROC curves for 1-, 2- and 3-year OS prediction of the prognostic signature in GSE21501 (C) and GSE62452 (H) cohorts. **(D and I)** PCA analysis based on the prognostic signature in GSE21501 (D) and GSE62452 (I) cohorts. **(E and J)** ROC curves of the risk score and other clinicopathological characteristics in GSE21501 (E) and GSE62452 (J) cohorts.

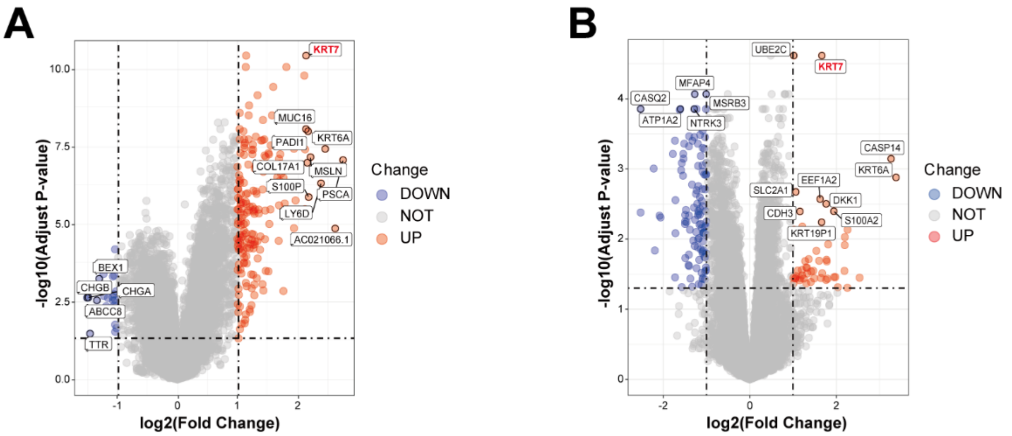

Fig.S3 Volcano plots of the DEGs between the high-risk and low-risk groups.

(A and B) Volcano plots of the DEGs between the high- and low-risk groups in TCGA (A) and ICGC (B) cohorts.

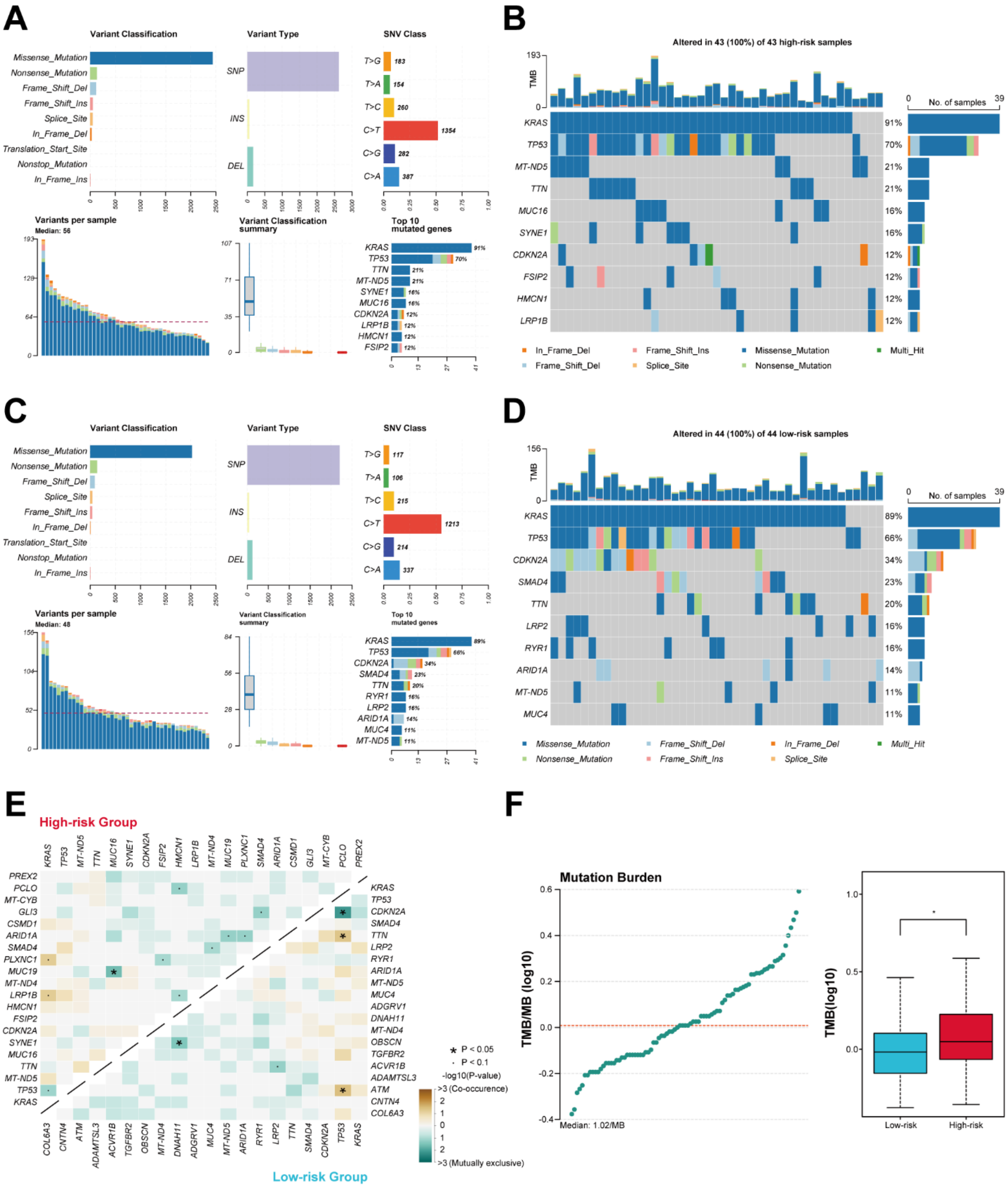

A
Variant Classification
Variant Type
SNV Class
Variants per sample
Median: 56
Variant Classification summary
Top 10 mutated genes
B
Altered in 43 (100%) of 43 high-risk samples
TMB
No. of samples
In_Frame_Del
Frame_Shift_Ins
Missense_Mutation
Multi_Hit
Frame_Shift_Del
Splice_Site
Nonsense_Mutation
C
Variant Classification
Variant Type
SNV Class
Variants per sample
Median: 48
Variant Classification summary
Top 10 mutated genes
D
Altered in 44 (100%) of 44 low-risk samples
TMB
No. of samples
E
High-risk Group
Low-risk Group
P < 0.05
P < 0.1
-log10(P-value)
>3 (Co-occurence)
>3 (Mutually exclusive)
F
Mutation Burden
TMB/MB (log10)
Median: 1.02/MB
TMB(log10)
Low-risk
High-risk

Fig.S4 Somatic mutation profiles between the high-risk and low-risk groups in ICGC cohorts.

(A−D) MAF-summary plots and waterfall charts of somatic mutations in the high-risk group (A and B) and low-risk group (C and D). The top 10 mutated genes are shown. **(E)** Correlation heatmaps of co-occurrence and mutually exclusive mutations in the high-risk and low-risk groups. **(F)** Distribution of TMB (left) and comparison between two risk groups (right). *, $P < 0.05$.

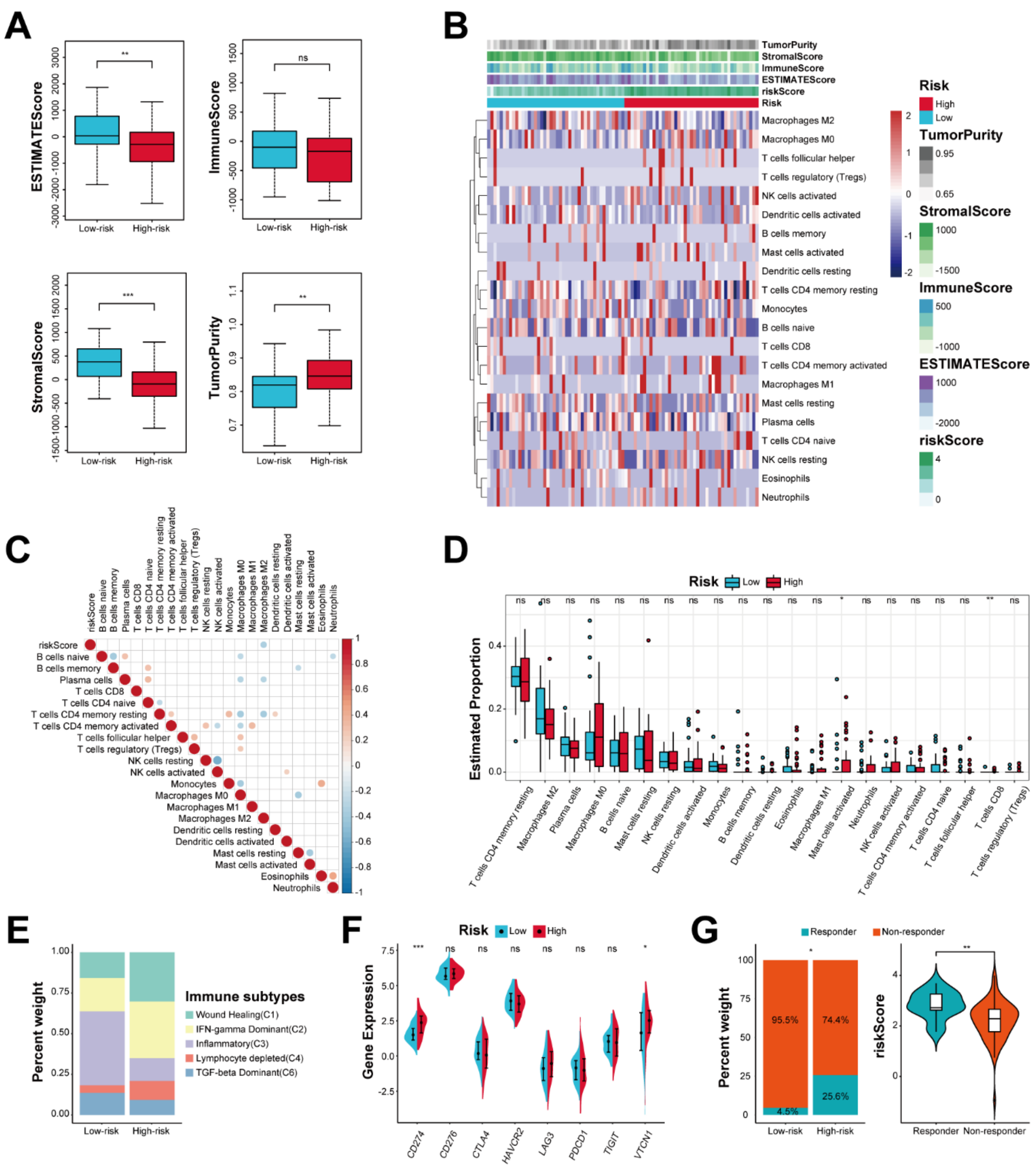

Fig.S5 Estimation of immune cell infiltration and prediction of ICB responses in ICGC cohort.

(A) Comparisons of estimate score, immune score, stromal score and tumor purity between the two risk groups. **(B)** Heatmap displaying the abundance of 21 different immune cells. **(C)** Correlation heatmap of different immune cells and the risk scores. **(D)** Comparison of CIBERSORT scores of immune cells between the two risk groups. **(E)** Proportions of five immune subtypes in two risk groups. **(F)** The expression levels of eight immune checkpoints in two risk groups. **(G)** Comparison of ICB

response rates between the two risk groups, and the risk score between responders and non-responders.

ns, not significant; *, $P < 0.05$; **, $P < 0.01$; ***, $P <0.001$.

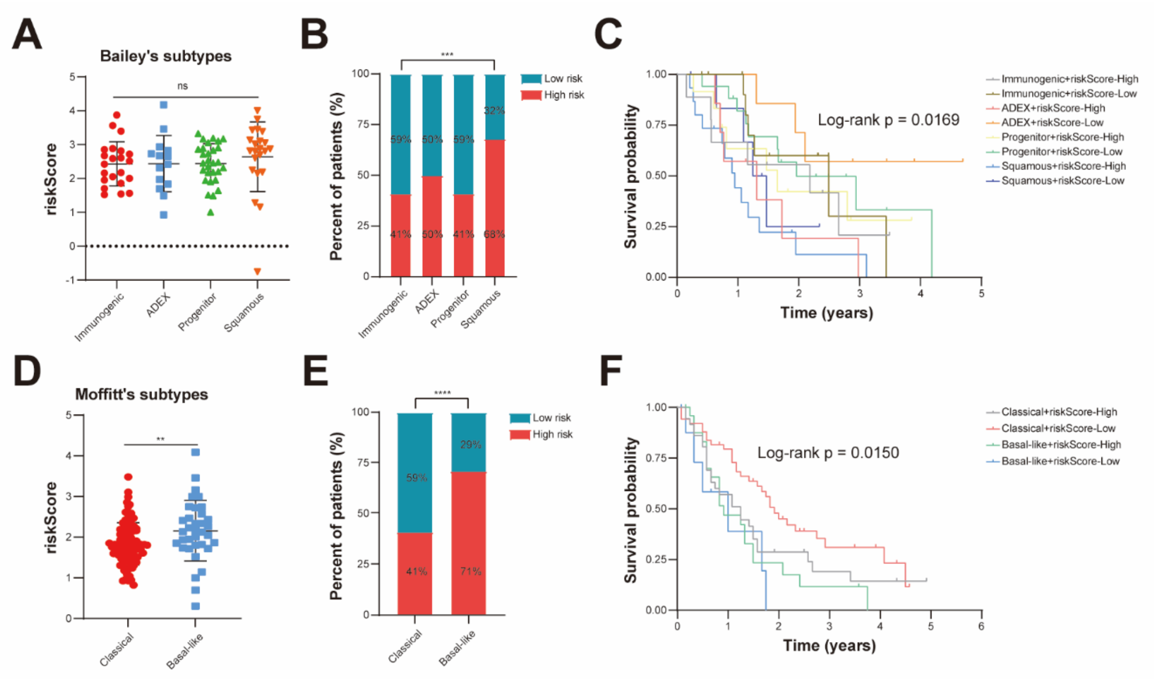

Fig.S6 The correlation between CIC-related prognostic signature and other molecular subtypes of PDAC.

(A) The comparison of CIC-related risk score in Bailey's subtypes (the expression and clinical data were obtained from ICGC dataset). **(B)** The proportion of patients from the high-risk group and low-risk group in Bailey's subtypes (patients were stratified into the low- and high-risk subgroups according to the median value of CIC-related risk score). **(C)** Kaplan-Meier survival analysis based on the combination of CIC-related risk score and Bailey's subtypes. **(D)** The comparison of CIC-related risk score in Moffitt's subtypes (the expression and clinical data were obtained from GSE71729 dataset). **(E)** The proportion of patients from the high-risk group and low-risk group in Moffitt's subtypes (patients were stratified into the low- and high-risk subgroups according to the median value of CIC-related risk score). **(F)** Kaplan-Meier survival analysis based on the combination of CIC-related risk score and Moffitt's subtypes. ns, no significant; **, *P* <0.01; ***, *P* <0.001; ****, *P* <0.0001.

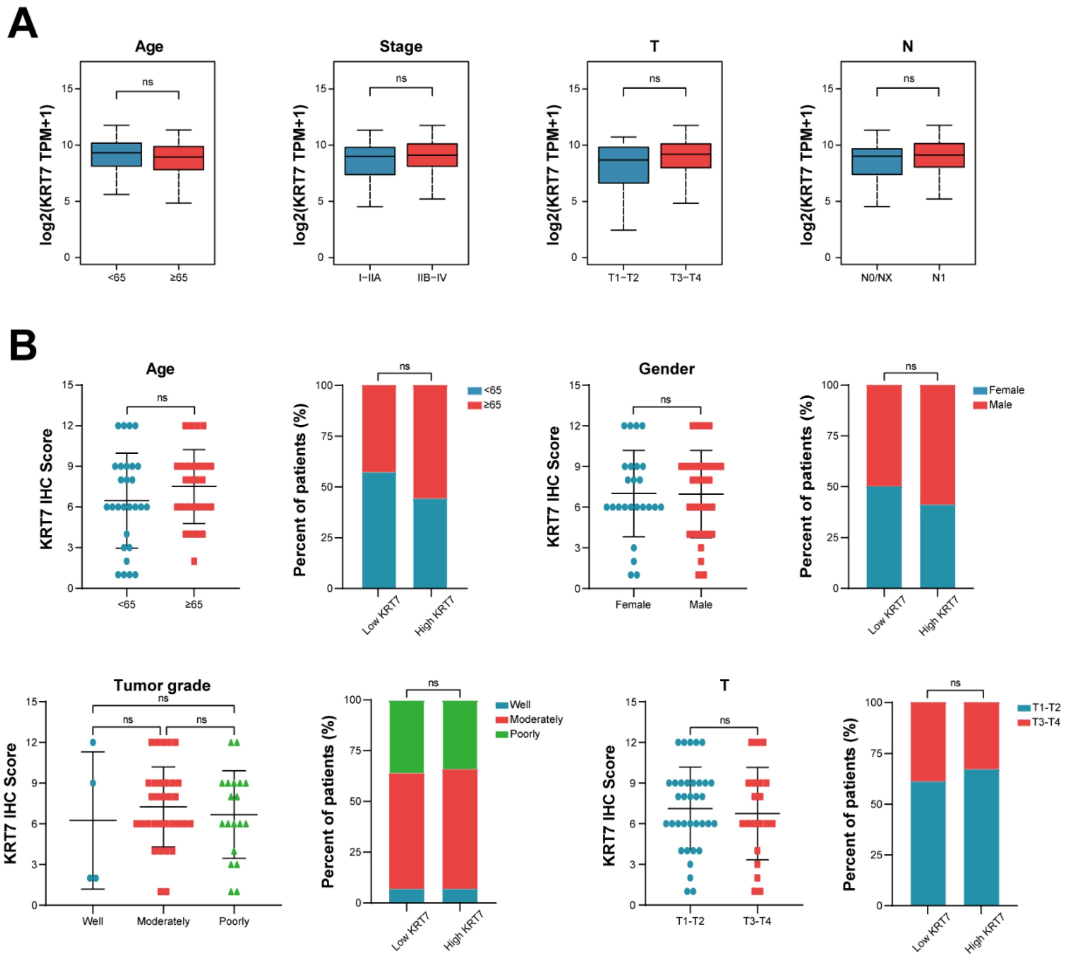

rr

Fig.S7 The correlation between KRT7 expression and clinicopathological characteristics.

(A) The comparison of *KRT7* expression between age, AJCC stage, T stage and N stage subgroups in TCGA cohort. **(B)** The comparison of KRT7 IHC scores between age, sex, tumor grade and T stage subgroups, and proportions of these characteristics between the KRT7 low- and high-expression subgroups in PUMCH cohort (n = 55). Low expression, IHC scores 1−6; high expression, IHC scores 8−12. Patients were stratified into the low- and high-expression groups based on the median IHC score (median value = 6). ns, not significant.

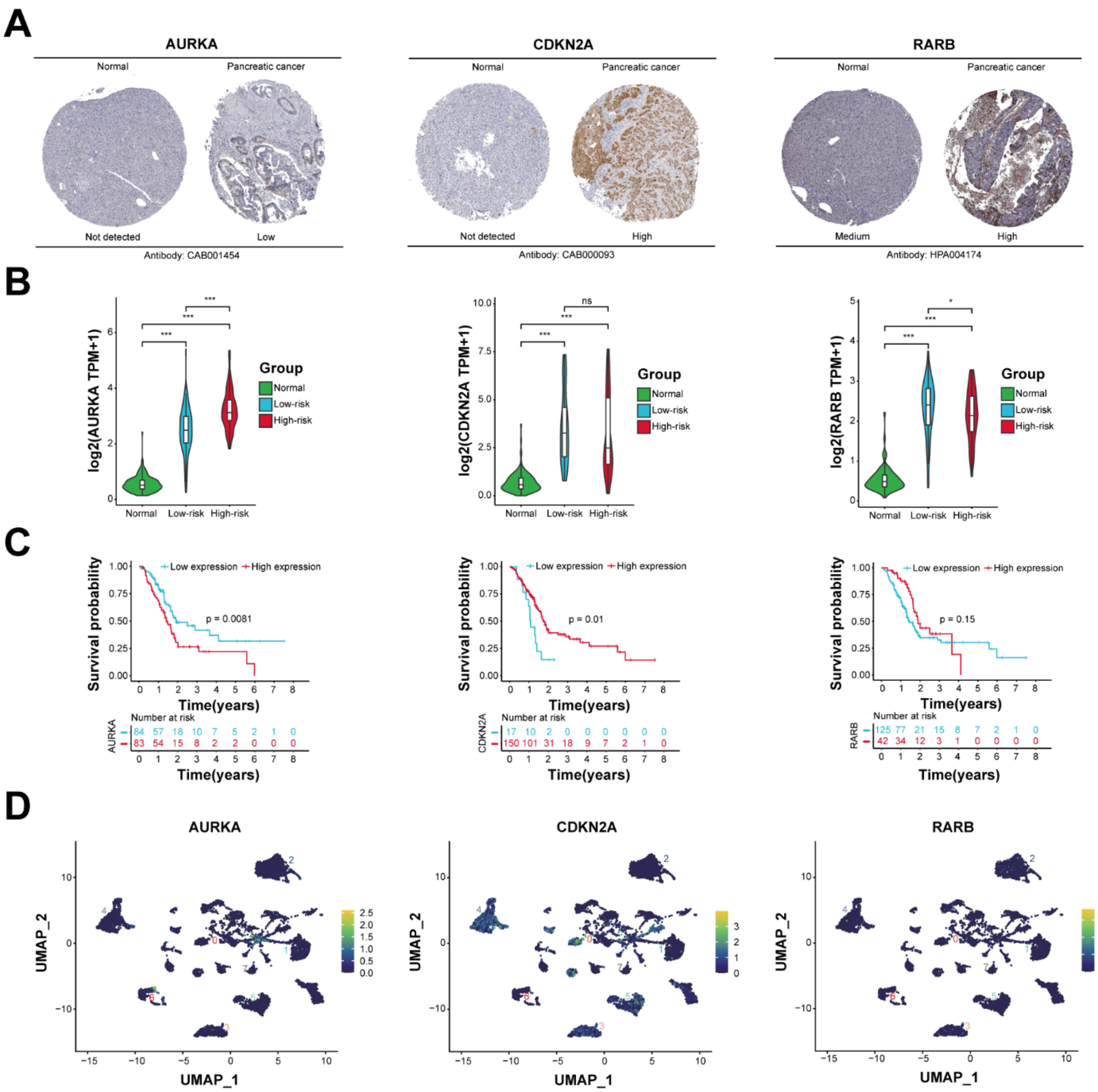

Fig.S8 Expression levels and survival analysis for *AURKA*, *CDKN2A* and *RARB*.

(A) Representative IHC images of AURKA, CDKN2A and RARB in normal and PC tissues on HPA database. **(B)** Comparison of *AURKA*, *CDKN2A* and *RARB* expression between normal samples (GTEx dataset) and tumor samples (TCGA dataset). TCGA patients were stratified into the low- and high-risk groups based on the risk score of individuals. **(C)** Kaplan-Meier survival analysis based on the median or optimal cut-off value of *AURKA*, *CDKN2A* and *RARB* expression in TCGA dataset. **(D)** Relative

expression of *AURKA*, *CDKN2A* and *RARB* among eight malignant clusters visualized by UMAP projection based on single-cell transcriptome analysis. ns, not significant; *, $P < 0.05$; ***, $P < 0.001$.

Table. S1 The list of 101 CIC-related genes.

	SYMBOL		SYMBOL		SYMBOL		SYMBOL
1	*APP*	31	*ERCC6*	61	*NTS*	91	*TGFB1*
2	*AR*	32	*ERI3*	62	*NUPR1*	92	*TM9SF4*
3	*ARHGAP36*	33	*EZR*	63	*PARD3*	93	*TNFSF10*
4	*ARHGEF11*	34	*FMN1*	64	*PCDH7*	94	*TP53*
5	*ATG5*	35	*FOS*	65	*PDPN*	95	*TP63*
6	*ATG7*	36	*GULP1*	66	*PIK3C3*	96	*TUBA1A*
7	*ATP6V1H*	37	*GZMB*	67	*PIKFYVE*	97	*UVRAG*
8	*AURKA*	38	*HTT*	68	*PRKAA2*	98	*VCL*
9	*BECN1*	39	*IL10*	69	*PRND*	99	*VIM*
10	*CALB2*	40	*IL6*	70	*PRNP*	100	*WT1*
11	*CAV1*	41	*ITGB1*	71	*PTK2*	101	*ZBTB38*
12	*CDC20*	42	*RUBCN*	72	*RAC1*		
13	*CDC42*	43	*KIF2C*	73	*RARB*		
14	*CDH1*	44	*KRT7*	74	*RASA2*		
15	*CDH3*	45	*LPAR2*	75	*RHOA*		
16	*CDKN2A*	46	*MAD2L1*	76	*RND3*		
17	*CEBPB*	47	*MAP1LC3A*	77	*RNF146*		
18	*CTNNA1*	48	*MAPK1*	78	*ROCK1*		
19	*CTNNB1*	49	*MAPT*	79	*ROCK2*		
20	*CTSB*	50	*MCOLN1*	80	*S100B*		
21	*CTSK*	51	*MLANA*	81	*SCN2A*		
22	*CTSL*	52	*MRTFA*	82	*SFTPB*		
23	*CTSS*	53	*MSMB*	83	*SNCA*		
24	*CTTN*	54	*MT2A*	84	*SOD1*		
25	*CUEDC2*	55	*MTMR7*	85	*SPRN*		
26	*CXCL8*	56	*MTOR*	86	*SRC*		
27	*CYBB*	57	*MTUS2*	87	*SRF*		
28	*DIAPH1*	58	*MYC*	88	*STMN2*		
29	*EEA1*	59	*MYL2*	89	*TF*		
30	*EGFR*	60	*MYLK*	90	*TFF3*		

Table. S2 Univariate and multivariate Cox regression analyses of the prognostic signature and clinicopathological factors.

	Univariate Cox Regression			Multivariate Cox Regression		
**Modeling cohort**	**Characteristics**	**HR (95%CI)**	***P* value**	**Characteristics**	**HR (95%CI)**	***P* value**
TCGA	Age (≥ 65 vs. < 65)	1.387 (0.912−2.109)	0.127	Age (≥ 65 vs. < 65)	1.273 (0.833−1.946)	0.264
	Gender (Male vs. Female)	0.877 (0.581−1.325)	0.534	Gender (Male vs. Female)	0.691 (0.449−1.064)	0.093
	Grade (G3−G4 vs. G1−G2)	1.483 (0.961−2.290)	0.075	Grade (G3−G4 vs. G1−G2)	1.064 (0.681−1.661)	0.786
	Stage (III−IV vs. I−II)	0.813 (0.256−2.580)	0.726	Stage (III−IV vs. I−II)	1.439 (0.427−4.844)	0.557
	T (T3−T4 vs. T1−T2)	2.281 (1.176−4.421)	0.015	T (T3−T4 vs. T1−T2)	1.209 (0.608−2.403)	0.588
	N (N1 vs. N0/NX)	2.184 (1.311−3.638)	0.003	N (N1 vs. N0/NX)	1.790 (1.030−3.110)	0.039
	Risk Score	2.722 (1.905−3.889)	< 0.001	Risk Score	2.935 (1.977−4.357)	< 0.001
**Validation cohort**						
ICGC	Age (≥ 65 vs. < 65)	1.263 (0.733−2.178)	0.400	Age (≥ 65 vs. < 65)	1.674 (0.932−3.008)	0.085
	Gender (Male vs. Female)	1.091 (0.638−1.867)	0.750	Gender (Male vs. Female)	1.021 (0.585−1.783)	0.942
	Grade (G3−G4 vs. G1−G2)	2.289 (1.326−3.951)	0.003	Grade (G3−G4 vs. G1−G2)	2.105 (1.158−3.826)	0.015
	T (T3−T4 vs. T1−T2)	1.692 (0.759−3.770)	0.199	T (T3−T4 vs. T1−T2)	1.987 (0.848−4.652)	0.114
	N (N1 vs. N0/NX)	2.733 (1.399−5.339)	0.003	N (N1 vs. N0/NX)	2.978 (1.458−6.081)	0.003

	Risk Score	1.619 (1.098−2.388)	0.015	Risk Score	1.924 (1.250−2.962)	0.003
GSE21501	T (T3−T4 vs. T1−T2)	0.948 (0.514−1.749)	0.864	T (T3−T4 vs. T1−T2)	0.565 (0.297−1.077)	0.083
	N (N1 vs. N0/NX)	5.910 (2.096−16.661)	0.001	N (N1 vs. N0/NX)	5.865 (2.027−16.969)	0.001
	Risk Score	1.777 (1.214−2.601)	0.003	Risk Score	1.611 (1.093−2.374)	0.016
GSE62452	Grade (G3−G4 vs. G1-G2)	2.151 (1.177−3.933)	0.013	Grade (G3−G4 vs. G1-G2)	1.605 (0.834−3.087)	0.157
	Stage (III−IV vs. I−II)	1.320 (0.704−2.477)	0.387	Stage (III−IV vs. I−II)	1.144 (0.604−2.166)	0.679
	Risk Score	3.662 (1.777−7.544)	< 0.001	Risk Score	2.972 (1.386−6.373)	0.005

Supplementary material

Unprocessed western blots of Figure. 9B

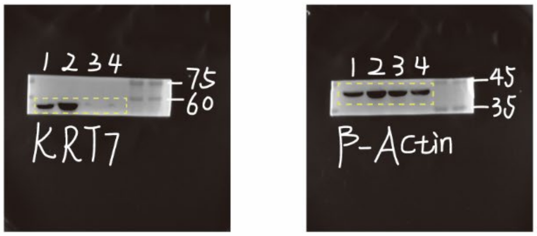

1. BxPC-3
2. CFPAC-1
3. PANC-1
4. MIA PaCa-2

Unprocessed western blots of Figure. 10A

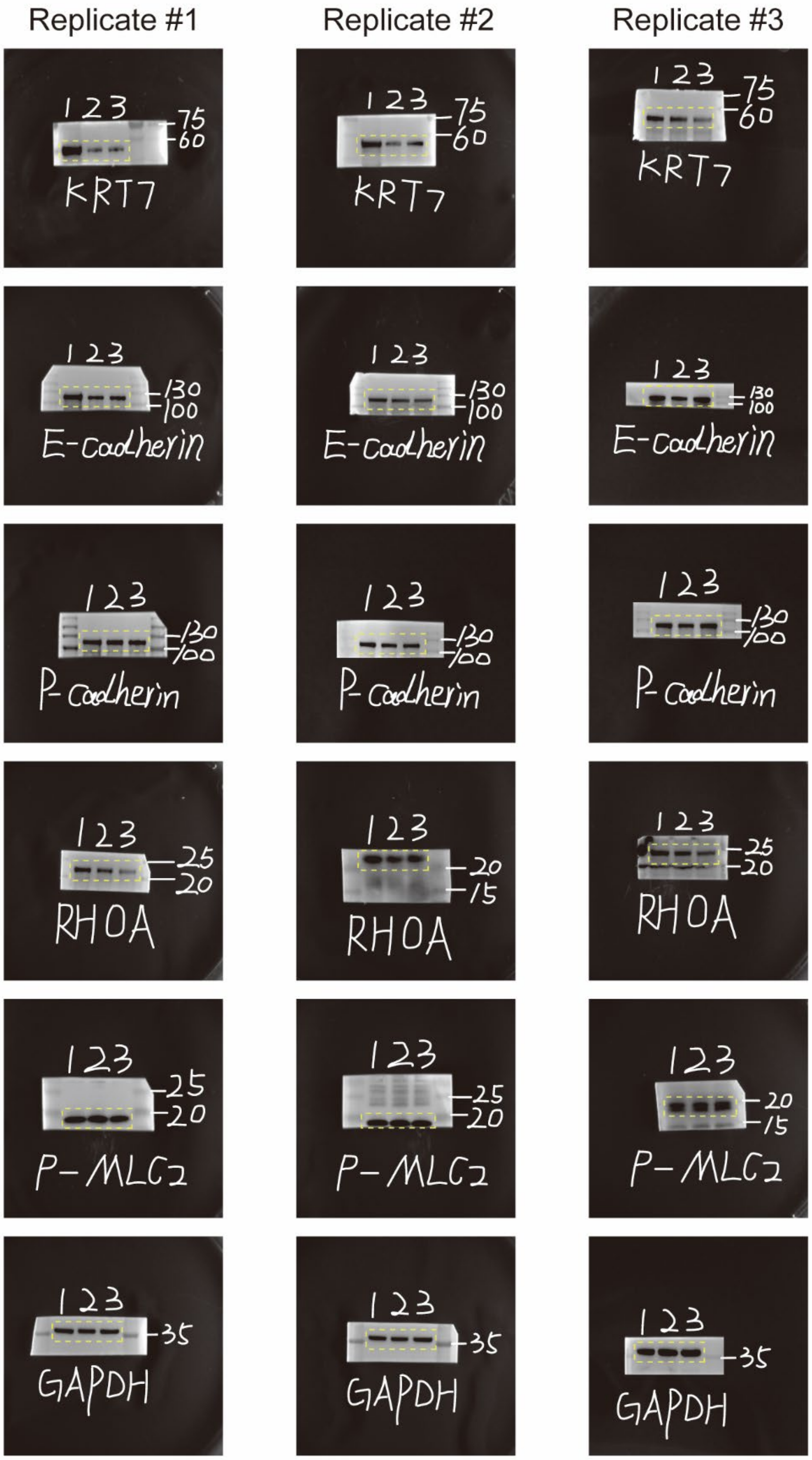

BxPC-3

1. siControl

2. siKRT7#1

3. siKRT7#2

Unprocessed western blots of Figure. 10A

Replicate #1 Replicate #2 Replicate #3

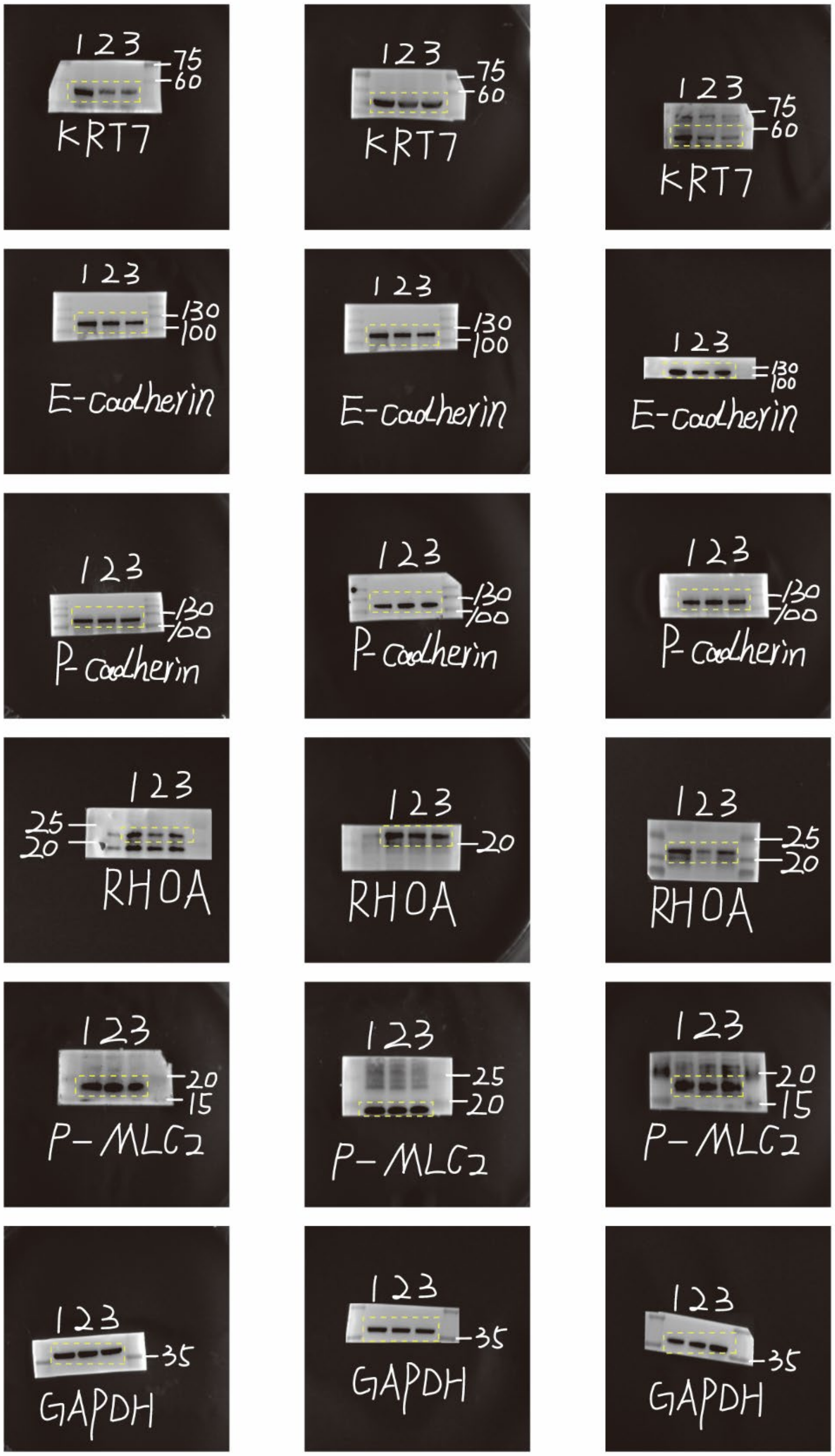

CFPAC-1

1. siControl

2. siKRT7#1

3. siKRT7#2
